# A practical introduction to using the drift diffusion model of decision-making in cognitive psychology, neuroscience, and health sciences

**DOI:** 10.3389/fpsyg.2022.1039172

**Published:** 2022-12-09

**Authors:** Catherine E. Myers, Alejandro Interian, Ahmed A. Moustafa

**Affiliations:** ^1^Research and Development Service, VA New Jersey Health Care System, East Orange, NJ, United States; ^2^Department of Pharmacology, Physiology and Neuroscience, New Jersey Medical School, Rutgers University, Newark, NJ, United States; ^3^Mental Health and Behavioral Sciences, VA New Jersey Health Care System, Lyons, NJ, United States; ^4^Department of Psychiatry, Robert Wood Johnson Medical School, Rutgers University, Piscataway, NJ, United States; ^5^Department of Human Anatomy and Physiology, The Faculty of Health Sciences, University of Johannesburg, Johannesburg, South Africa; ^6^School of Psychology, Faculty of Society and Design, Bond University, Robina, QLD, Australia

**Keywords:** drift diffusion model, speed-accuracy tradeoff, computational model, decision making, evidence accumulation model, reaction time

## Abstract

Recent years have seen a rapid increase in the number of studies using evidence-accumulation models (such as the drift diffusion model, DDM) in the fields of psychology and neuroscience. These models go beyond observed behavior to extract descriptions of latent cognitive processes that have been linked to different brain substrates. Accordingly, it is important for psychology and neuroscience researchers to be able to understand published findings based on these models. However, many articles using (and explaining) these models assume that the reader already has a fairly deep understanding of (and interest in) the computational and mathematical underpinnings, which may limit many readers’ ability to understand the results and appreciate the implications. The goal of this article is therefore to provide a practical introduction to the DDM and its application to behavioral data – without requiring a deep background in mathematics or computational modeling. The article discusses the basic ideas underpinning the DDM, and explains the way that DDM results are normally presented and evaluated. It also provides a step-by-step example of how the DDM is implemented and used on an example dataset, and discusses methods for model validation and for presenting (and evaluating) model results. Supplementary material provides R code for all examples, along with the sample dataset described in the text, to allow interested readers to replicate the examples themselves. The article is primarily targeted at psychologists, neuroscientists, and health professionals with a background in experimental cognitive psychology and/or cognitive neuroscience, who are interested in understanding how DDMs are used in the literature, as well as some who may to go on to apply these approaches in their own work.

## Introduction

An important domain in cognitive psychology and cognitive neuroscience is decision-making: the process of recognizing features of the situation in which we find ourselves, considering numerous possible alternative responses, selecting and executing one response, observing the outcomes, and adjusting our behavior accordingly. Disruption to any of these processes can affect decision-making, with real-world consequences; examples include addiction, where individuals make decisions to sacrifice long-term health for short term benefits of the addictive substance or behavior (Bechara, 2005; Balodis and Potenza, 2020), but abnormal decision-making has also been implicated in disorders ranging from depressive and anxiety disorders (Chen, 2022) to borderline personality disorders (Hallquist et al., 2018) to Parkinson’s disease (Frank et al., 2007; Moustafa et al., 2008) to suicidality (Jollant et al., 2011; Brenner et al., 2015; Dombrovski and Hallquist, 2017). Better understanding of the cognitive and brain substrates of abnormal decision-making in these populations is key to improving both psychological and pharmacological treatments as well as treatment adherence.

One approach to understanding decision-making is through computational models, such as the drift diffusion model (DDM; Ratcliff, 1978; Ratcliff and McKoon, 2008; Ratcliff et al., 2016), which was originally developed to describe how well-trained participants make rapid decisions between two possible response alternatives. Such computational models attempt to impute information about latent cognitive processes based on observable decision-making behavior. By providing a mathematical framework to describe behavior, computational models can allow researchers to make explicit the underlying mechanistic processes that give rise to observable actions (Montague et al., 2012; Millner et al., 2020).

Although first described over 50 years ago, the DDM has recently enjoyed widespread use, partly due to the development of powerful and freely-available software implementing computationally-intensive model-fitting algorithms, and partly due to an accumulating literature documenting that the DDM can indeed shed light on latent cognitive processes that are not necessarily evident from traditional hypothesis-driven methods of behavioral data analysis (Deghan et al., 2022), and that have been linked to specific brain regions (Mulder et al., 2012; Mueller et al., 2017; Weigard and Sripada, 2021; Gupta et al., 2022).

The DDM is thus of broad interest in cognitive psychology and cognitive neuroscience (Evans and Wagenmakers, 2020), which has led to a burgeoning literature including many primary research reports that use the DDM to complement traditional statistical analysis of the behavioral data. Unfortunately, most if not all such articles assume readers’ familiarity with modeling jargon and graphical conventions (such as “parameter recovery studies,” “non-decision times,” and “hairy caterpillars”), hindering the ability of many readers to fully understand these results and their implications. Our own experience in different research institutions has suggested that many in the fields of psychology and neuroscience are somewhat intimidated by unfamiliar computational models, or by the time and effort that seems to be required to understand these models and their use.

The current article thus aims to provide a reader-friendly introduction to the DDM and its use. The article discusses the basic ideas underpinning the DDM, provides a step-by-step example of how the DDM is implemented and used on an example dataset, and discusses methods for model validation and conventions for presenting (and evaluating) model results. The goal is to provide sufficient background for a reader to critically read and evaluate articles using the DDM – without necessarily mastering the detailed mathematical underpinnings. However, for those readers who wish to go a little deeper, the Supplemental Material provides R script to allow readers to run the examples discussed in the text, and to generate the data figures and tables shown in the article (see Appendix).

Importantly, the current article is not meant to offer a comprehensive review of the DDM literature (for good reviews, see, e.g., Forstmann et al., 2016; Ratcliff et al., 2016; Evans and Wagenmakers, 2020; Gupta et al., 2022), nor a general tutorial on good computational modeling practices (see, e.g., Daw, 2011; Heathcote et al., 2015; Wilson and Collins, 2019); however, it may provide a useful springboard for cognitive psychologists and neuroscientists considering the use of the drift diffusion model, and related computational models, in their own research.

## Overview of the drift diffusion model

Speeded decision-making tasks in cognitive psychology require well-trained participants to rapidly choose between two or more competing responses. Examples include lexical decision tasks (press one key if the stimulus is a word or another if it is a non-word), Stroop tasks (press a key corresponding to the color in which a word is printed, ignoring the semantic content), and saccadic flanker tasks (move the eye in the direction indicated by a central stimulus, ignoring the directionality of flanker stimuli).

On such tasks, even well-trained participants show a speed-accuracy tradeoff: they may increase accuracy at the expense of slower (more careful) responding, or make very quick decisions that are more likely to be erroneous (Schouten and Bekker, 1967; Wickelgren, 1977). This speed-accuracy tradeoff appears to be at least partially under conscious control, because a participant can perform differently when instructed to emphasize speed vs. accuracy (Ratcliff and Rouder, 1998; Voss et al., 2004; Milosavljevic et al., 2010; Katsimpokis et al., 2020). This complicates the interpretation of behavioral data. Additionally, differences in response time across groups might reflect different underlying mechanisms (Voss et al., 2013). For example, two patient groups might both have slower mean reaction times (RTs) than a healthy comparison group, but in one patient group this might reflect more cautious decision-making, and in the other it might reflect disease-related slowing of motor responses. Ideally, what is needed is a way to evaluate data that considers not only accuracy and speed, but the interaction between them.

To address these issues, a complementary approach to analyzing the observed behavioral data is to use computational models that attempt to extract latent cognitive parameters that, together, could produce the observed distribution of RT and accuracy data.

The drift diffusion model (DDM), first described by Ratcliff and colleagues (Ratcliff, 1978; Ratcliff and McKoon, 2008; Ratcliff et al., 2016), is one example of a broader class of models called *evidence accumulation models*. These models conceptualize decision-making as a process in which, on each trial, individuals accumulate evidence favoring one or another possible response, until enough evidence accumulates to reach a criterion or threshold, at which point a decision is made and the corresponding response is initiated. Evidence accumulation models are sometimes called *sequential sampling models*, reflecting the idea that the nervous system repeatedly (sequentially) obtains bits of information (samples) from the environment, until a threshold of evidence is reached. The speed-accuracy tradeoff reflects a balance point determining when to stop sampling the environment and make a decision based on the data at hand.

Like all computational models, the DDM is defined by a series of mathematical equations, containing a number of *parameters*, that can be assigned different values. An easy way to think of parameters is as dials (or control bars) on a stereo system that each control one aspect of the sound (e.g., treble, bass, volume), and can be adjusted individually so that together they result in the desired effect. Similarly, parameters in an evidence accumulation model may control aspects such as how fast evidence is accumulated, a built-in bias for one response alternative over another, and a tendency to emphasize speed or accuracy. Each of these parameters can be adjusted in the model, affecting how the model behaves.

**Figure 1:**
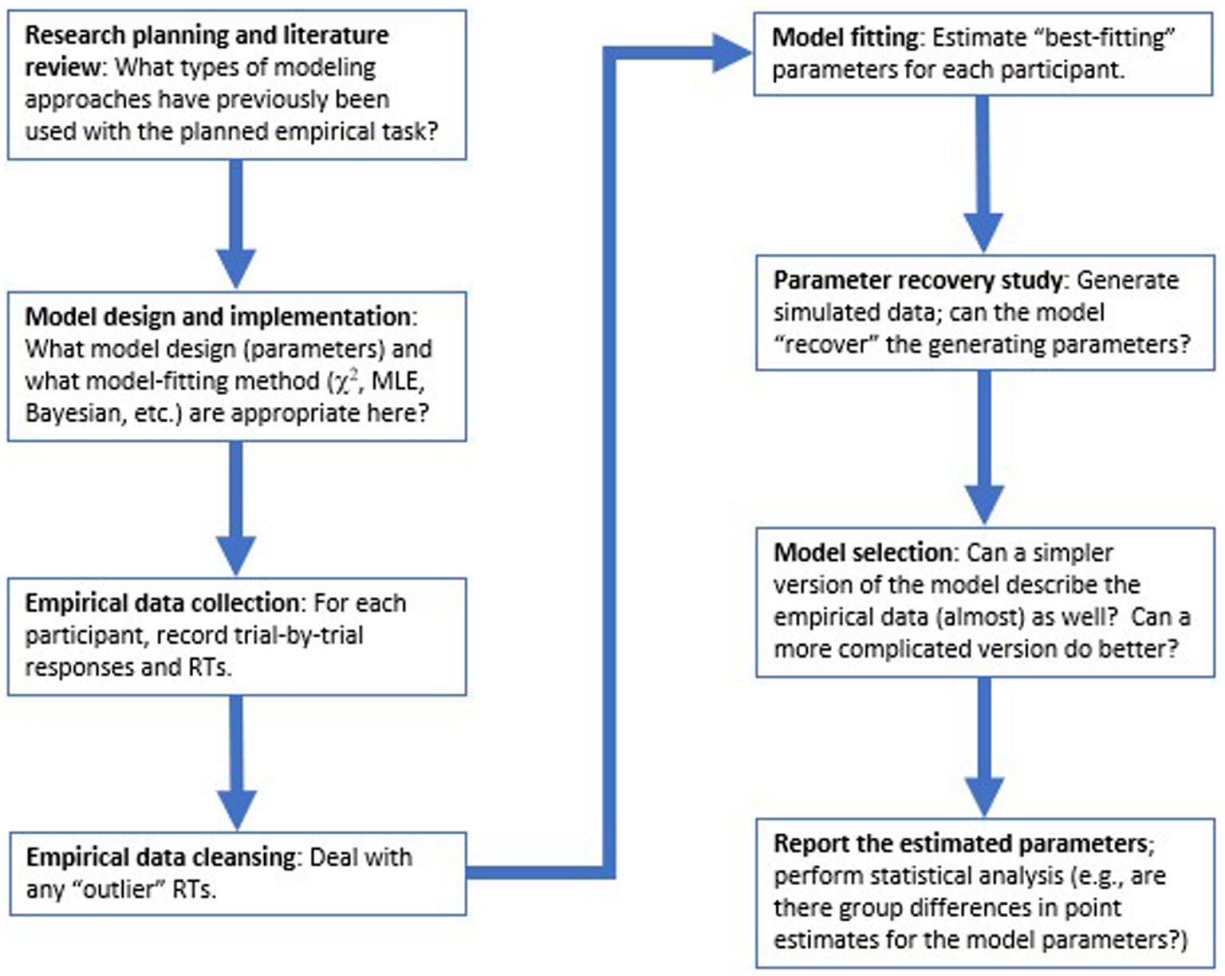
Overview of key steps in the modeling process. Typically, choices relating to model design and implementation should be made at task design stage, before collection of empirical data. Then, after data collection and data cleansing, model-fitting is conducted to estimate “best-fitting” parameters for each participant that allow the model to most closely replicate that participant’s accuracy and reaction time (RT) distributions, followed by model validation and model selection procedures, before the modeling results are reported and subjected to conventional statistical analysis.

In the sections below, we walk through major steps in the modeling process, following the flow-chart in Figure 1. First, the remainder of this section provides a high-level description of the DDM, its parameters, and its use in cognitive psychology. Then we consider a concrete example of how the DDM might be applied to a simple two-choice decision-making task, and step through the process of model-fitting to estimate parameter values for each participant in our dataset, followed by model validation and model selection approaches. Finally, we discuss how the model results can be reported and subjected to statistical analysis. We conclude with some thoughts about evaluating published research using the DDM and other computational models.

### Parameters in the drift diffusion model

**Figure 2:**
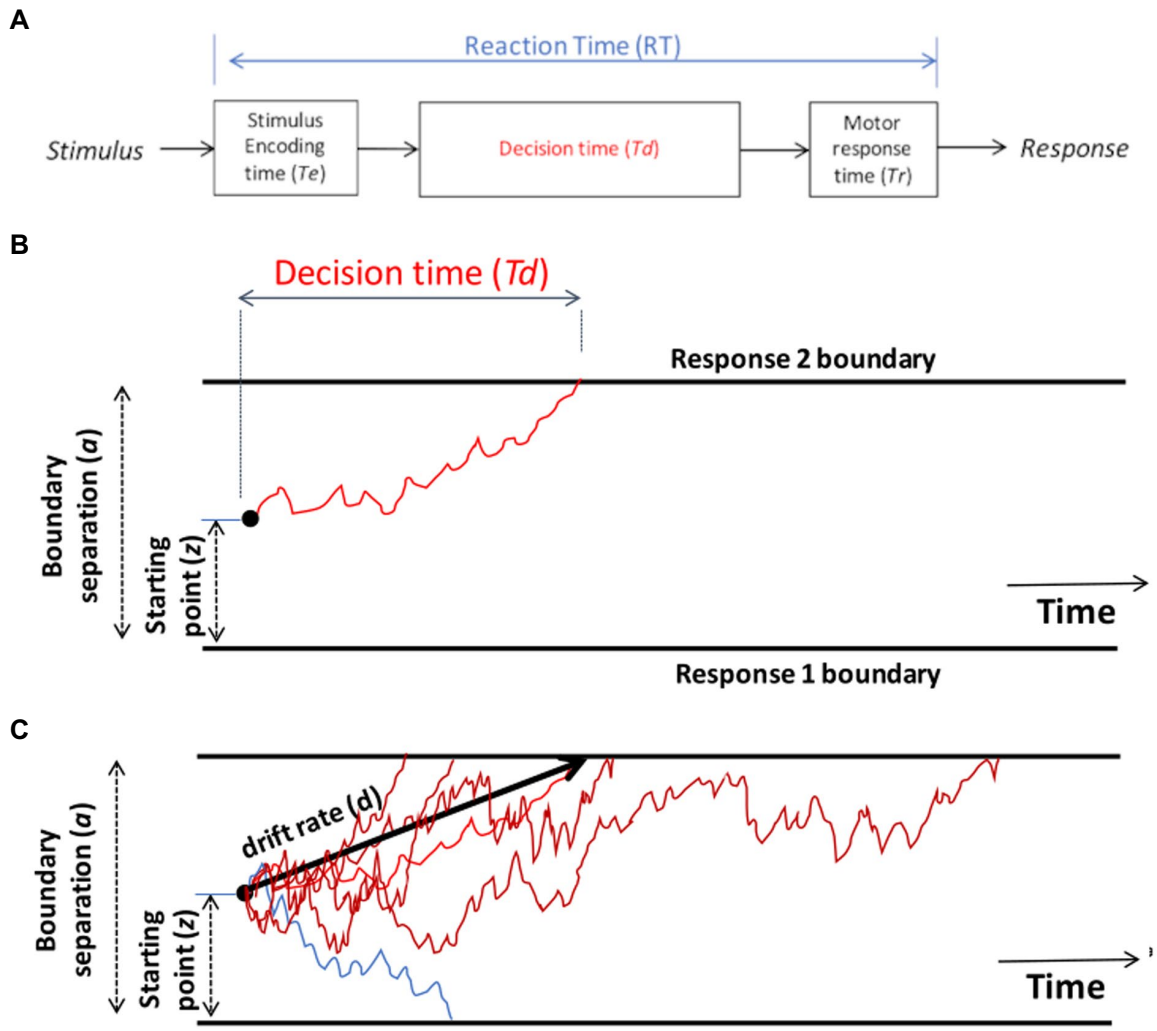
Schematic of the drift diffusion model (DDM). **(A)** Total reaction time (RT) on each trial is assumed to reflect the time required for the nervous system to encode the stimulus (*Te*), the time to make a decision (*Td*), and the time to execute the selected motor response (*Tr*). The encoding and response time are typically combined into a single parameter, *Ter*, representing non-decision time on each trial, so that RT = *Ter* + *Td*. **(B)** On each trial, the DDM assumes a process of noisy evidence accumulation, represented here as a red line, traveling from a starting point (*z*) toward boundaries representing two possible responses. When the decision-making process encounters one of the boundaries, the corresponding response is triggered, and the time to reach that boundary is the decision time *Td* for that trial. In this schematic, the upper boundary is crossed and Response 2 is chosen. The separation between the two boundaries (*a*) is one factor determining *Td*: the greater the boundary separation, the further the evidence accumulation process has to travel to reach a boundary, leading to longer decision times on average. The starting point *z* also influences *Td*: if *z* is placed closer to one boundary, it is easier to reach that boundary than the opposite boundary, leading to a response bias favoring the nearer boundary. **(C)** Schematic of the DDM decision-making process for several trials on which the correct response is Response 2 (upper boundary). Noise in the evidence accumulation process means that trials with the same stimulus may have different *Td* (and hence different RT), represented here as various red lines, and may on occasion even reach the opposite boundary, triggering an incorrect response, represented here as a blue line. The drift rate (*d*) is the average slope of the evidence accumulation process across a large number of trials.

The DDM starts with the assumption that the RT on each trial, defined as the time from stimulus onset until execution of the motor response, can be decomposed into three parts (Figure 2A): the time required for the nervous system to detect or *encode* the stimulus (often denoted *Te*), the time to reach a *decision* about how to respond to that stimulus (*Td*), and the time required for the nervous system to execute the chosen motor *response* (*Tr*). Thus, on a given trial, the observed reaction time is the sum of these three components: RT = *Te* + *Td* + *Tr*.

Although it might in principle be possible to measure *Te* and *Tr* separately, normally the encoding and response time are lumped together into a single parameter representing non-decision time (*Ter*): that portion of the RT that occurs independently of the decision-making process *Td*. Given this simplification, RT = *Ter* + *Td*. Typical values of *Ter* lie in the range 0.1–0.5 s, partly depending on the complexity of stimuli and the specific motor responses involved (e.g., people can generally execute saccades faster than keypresses). It’s usually assumed that *Ter* may differ across individuals, but is relatively constant across trials for one individual performing one task.

The other component of RT is decision time (*Td*), which is the time to make a decision (after the stimulus is encoded, but before the chosen response is executed). On a single trial, noisy information is accumulated across time while the process travels along a corridor bounded by the two possible responses (schematized by red line Figure 2B). As progressively more information is accumulated, evidence in favor of one response will “push” the decision process closer to the corresponding boundary. When one of the boundaries is reached, the corresponding response is selected, and time to reach that boundary defines the decision time *Td* on that trial.

By convention, the lower boundary is assigned a value of 0 on the *y*-axis and distance to the upper boundary is defined by a parameter representing boundary separation (*a*). Larger values of *a* mean that the decision-making process must travel further (up or down) to reach a boundary. The effect of larger *a* is thus that decision-making will be slower and more cautious: slower because more evidence is required before a distant boundary is reached and a response is triggered, and higher accuracy because it will be rare for the decision process to “mistakenly” cross the wrong boundary (Lerche et al., 2020). Boundary separation is in arbitrary units, but is often assumed to range from about 0.5–2. It is often assumed that the degree of boundary separation is at least partly under conscious control, depending on whether there is an emphasis on speed (low *a*) or accuracy (high *a*).

On each trial, the decision process starts from a location on the y-axis defined by a parameter denoting a relative starting point (*z*) that ranges from 0 (lower axis) to 1 (upper axis). If *z* = 0.5, the starting point is equidistant from the two boundaries. However, if *z* approaches 1 (or 0), the decision process starts off close to the upper (or lower) boundary on every trial, meaning that less information is required in order to reach that boundary and initiate the corresponding response. The starting point *z* therefore reflects a response bias in favor of one or the other response.

The decision-making process in the DDM is assumed to be noisy (schematized by the jagged red line in Figure 2B), reflecting noisy sensory inputs, stochastic variation in the firing rate of neurons in the decision-making centers of the brain, and even momentary fluctuations in attention. This noise means that the same stimulus may not generate the same decision time, or even the same response, every time it occurs – leading to variations in RT and response accuracy across trials; multiple trials with different decision times *Td* are schematized as multiple red lines in Figure 2C. Across many such trials, the average rate at which evidence accumulates toward the correct boundary is defined by a parameter denoting drift rate (*d*), schematized as the slope of the heavy black line in Figure 2C. Drift rate is a measure of speed of information processing, which may vary depending on task difficulty. For easy tasks with highly discriminable stimuli, there should be a high drift rate (steep slope up or down), and the evidence should accumulate quickly and reliably toward the correct boundary, resulting in fast RTs and high accuracy. For more difficult tasks or more ambiguous stimuli, the drift rate may be lower (less steep), meaning that evidence accumulation is slower and noisier, resulting in slower and more variable RTs.

As summarized in Table 1, then, the parameters of the DDM map onto different cognitive processes: speed-accuracy settings (boundary separation *a*), response bias (starting point *z*), information processing speed (drift rate *d*), and non-decision time (*Ter*). These parameters are sometimes called “free parameters,” in the sense that they can take on different values (“freely”) – and just like the knobs on a stereo, changing each parameter affects DDM behavior.

**Table 1:** Free parameters in a “standard” drift diffusion model (DDM), and associated latent cognitive processes.

DDMparameter	Parameter name	Typical range of values	Cognitive processes
*a*	Boundary separation	0.5–2 (in arbitrary units)	Response caution: higher *a* emphasizes accuracy over speed, lower *a* emphasizes speed over accuracy.
*z*	Starting point	0…1 (as proportion of *a*)	Response bias: starting point nearer to one boundary leads to faster and more common decisions favoring that response.
*d*	Drift rate	−5…+5 (values <0 slope down to lower boundary)	Speed of evidence accumulation processing: can be affected by task difficulty, stimulus discriminability, attention.
*Ter*	Non-decision time	0.1–0.5 s (cannot exceed total RT)	Neurological processes for registering (encoding) sensory stimuli and for executing motor responses.

Naming conventions for these parameters sometimes vary across software packages; for consistency, the above parameter names are used throughout this article.

**Figure 3:**
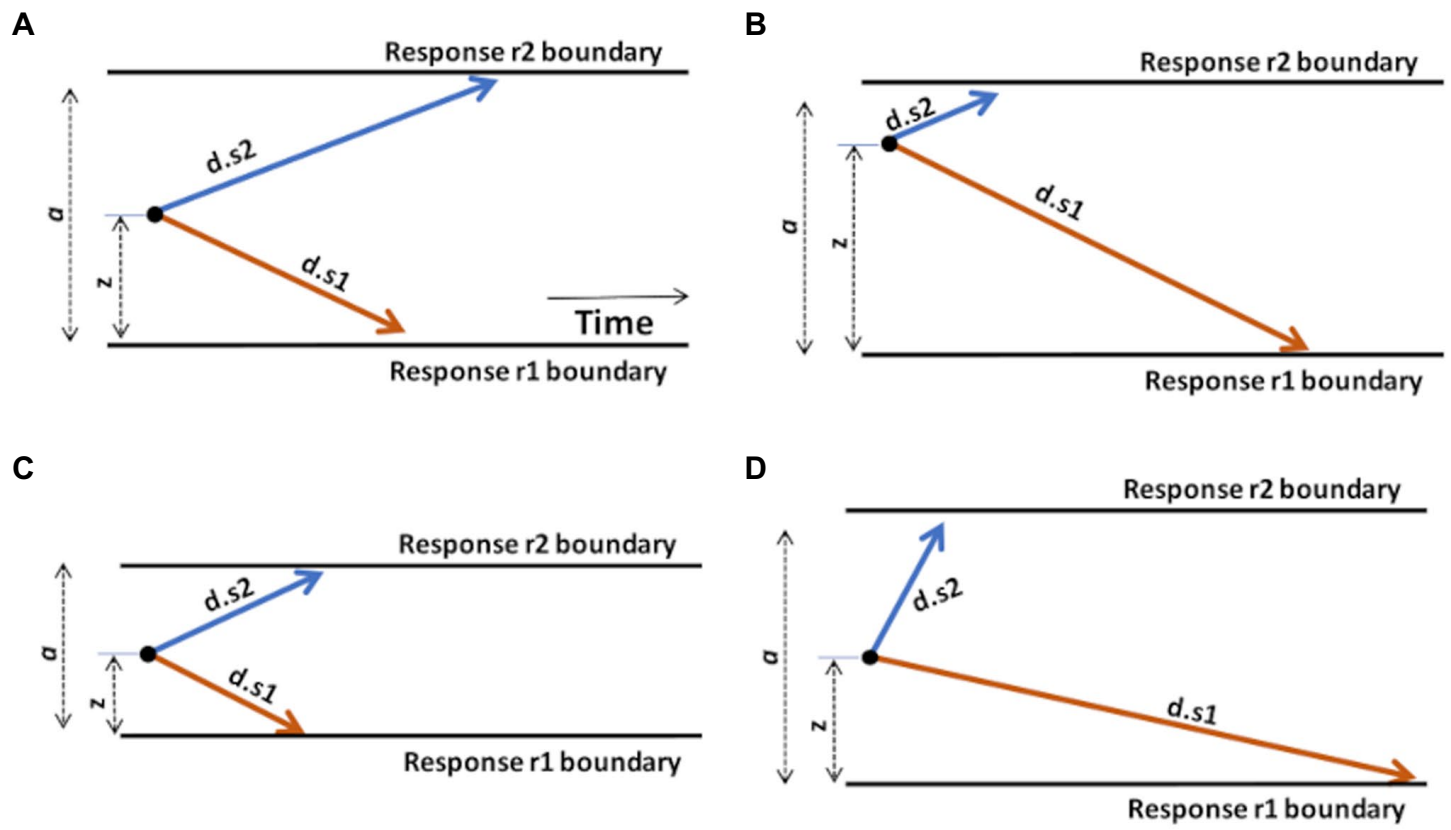
Graphical illustration of the effects of changing DDM parameters. **(A)** A “standard” DDM model, defined by boundary separation a, relative starting point *z*, and two drift rates *d.s1* and *d.s2* for trials associated with stimulus s1 or s2, respectively. The remaining parameter, non-decision time (*Ter*), is not shown but is assumed to represent a fairly constant contribution to overall RT, independent of the decision-making processes. Increasing or decreasing *Ter* will affect average RT regardless of other task considerations. **(B)** Changing the starting point *z*, by moving it closer to one boundary, introduces a response bias. Here, *z* moves closer to the upper boundary; on each trial, the evidence accumulation process has farther to go to reach r1 than r2, creating a response bias favoring r2 responses. **(C)** Reducing boundary separation a reduces the distance that the evidence accumulation process has to travel to cross a boundary. This will tend to result in faster responses and potentially more errors, since it is easier for noise to push the evidence accumulation process across the wrong boundary. Reduced a is therefore interpreted as reduced response caution: less evidence required before selecting either response. Increasing a has the opposite effect of increasing response caution: more evidence will be required before the evidence accumulation process crosses either boundary. **(D)** Increasing drift rate (here, *d.s2*) means that the evidence accumulation process will travel more quickly (steeply) toward the corresponding boundary: evidence accumulation is more efficient and, in effect, the task condition is easier. As a result, RTs on s2 trials will generally be faster. Decreasing a drift rate (here, *d.s1*) has the opposite effect: evidence accumulation on s1 trials is less efficient and the process proceeds more slowly toward the r1 boundary. As a result, RTs on s1 trials will generally be slower. Manipulating any model parameter individually can thus affect both accuracy and speed; model-fitting procedures find the configuration of parameter values (*a*, *z*, *d.s1*, *d.s2*, and *Ter*) that together provide the most accurate description of the observed accuracy and RT distributions.

For example, let us consider a task, schematized in Figure 3A, in which the subject is instructed to execute one response r1 as quickly as possible whenever stimulus s1 is shown, but a different response r2 whenever stimulus s2 is shown.

As shown in Figure 3B, increasing the starting point (*z*) will move the starting point closer to the upper boundary, meaning that the evidence accumulation process has farther to travel to reach the r1 boundary than to the r2 boundary, making it easier (and faster) to decide in favor of r2 on any trial. Such a prepotent response bias for r2 might be created if, say, r2 responses are much more frequent or highly-rewarded in the task.

As shown in Figure 3C, decreasing the boundary separation (*a*) would make both responses faster, without necessarily favoring one over the other. It also increases the error rate, because it’s easy to for noise to push the decision-making process across either boundary. A reduced boundary separation might happen if, say, the subject had been instructed to respond quickly, even at the expense of reduced accuracy. Increasing *a* would have the opposite effect of increasing response caution and producing slower RTs.

As shown in Figure 3D, increasing the drift rate for one type of stimulus (here, *d.s2*) would result in faster evidence accumulation on s2 trials, while decreasing the other (here, *d.s1*) would result in slower evidence accumulation on s1 trials. Drift rates are typically slower (less steep) under more difficult task conditions, with decreased stimulus discriminability, or in the presence of distracting stimuli.

Finally, increasing or decreasing non-decision time *Ter* would affect overall RT, without otherwise affecting the decision-making process. For example, patients with motor dysfunction might have increased *Ter* (and overall RT), independent of decision-making considerations.

Together, the values of the DDM parameters *Ter, a, z,* and *d* interact to affect overall task performance, including both accuracy and RT. To use the stereo example again, the auditory effect of changing one parameter (bass) may be very different depending on whether the value of another (volume) is low or high. To understand these complex interactions, rather than using schematics such as Figure 3, the DDM can be instantiated as a computer program, which includes equations describing the drift diffusion process, and specific values of each parameter *Ter, a, z,* and *d*. The model is then applied to the behavioral task: On each trial, the model is presented with a stimulus (e.g., s1 or s2), and the diffusion process starts from *z* and is allowed to run (in effect, creating a trace such as the ones shown in Figure 2C) until it crosses one or the other boundary at time *Td*, and triggers the corresponding motor response (r1 or r2), resulting in reaction time RT = *Ter* + *Td*. Over a series of such trials, usually the same stimuli in the same order as experienced by a human participant, the model’s responses and RTs are recorded, producing “simulated data.” The accuracy and RT distributions in the simulated data can then be compared against the accuracy and RT distributions in the empirical data, to see how well the model replicates or “fits” the empirical data. Typically, the goal is to fine-tune the parameter values until the model’s accuracy and RT distributions are as close as possible to the empirical data – a process called *model-fitting* or *parameter estimation*.

### Elaborations of the drift diffusion model

Before going on, it’s worth noting that the above description considers a “standard” DDM with four free parameters *Ter, a, z*, and *d*. More elaborate versions can be considered. For example, in many cases, it makes sense to consider different drift rates for different trial types or conditions. For example, a lexical decision task might have two types of stimuli (s1 = nonwords and s2 = words) but also have easy and hard trials, depending on whether the trigrams are visually degraded or not, or whether the non-words are pronounceable or not. In such cases, it may make sense to allow different drift rates for each combination of stimulus conditions, with the expectation that (for most participants), there will be a steeper drift rate for trials under the easy condition than the harder condition. In the schematic of Figure 3A, then, instead of having one drift rate for each stimulus (*d.s1* and *d.s2*), we might have one for each configuration of stimulus and condition (*d.s1.hard*, *d.s1.easy*, *d.s2.hard*, *d.s2.easy*).

Versions of the DDM have also been considered that include additional free parameters specifying the amount of trial-by-trial variation in the DDM parameters (Forstmann et al., 2016); however, simpler models ignoring this variability can often account for observed behavior as well as (and more parsimoniously than) more complex models (e.g., Dutilh et al., 2019).

### Drift diffusion model parameters correspond to latent cognitive processes

The purpose of performing model-fitting to estimate parameter values is to provide some insight into the underlying cognitive processes. These processes are latent in the sense that we cannot observe them directly, only impute them based on the participant’s pattern of behavior. These latent processes may be very important for understanding the cognitive neuroscience of decision-making. For example, they may map onto different brain systems, and may vary in principled ways in patient groups with different neuropsychological disorders.

In a computational model, we have the advantage that those cognitive processes are made explicit, in the form of the parameter values governing the model’s behavior. This can provide a way to identify specific cognitive mechanisms that underlie group differences.

A main reason for the recent popularity of the DDM is that the linkage between these DDM parameters and cognitive processes has been validated in a number of cognitive psychology studies, showing that changes in task conditions can alter DDM parameters in a principled way (e.g., Milosavljevic et al., 2010; Mulder et al., 2012). For example, participants instructed to “work especially carefully and avoid mistakes,” that is, to emphasize accuracy over speed, show larger boundary separation *a*, corresponding to greater response caution (Voss et al., 2004), while participants working under time pressure, i.e., emphasizing speed over accuracy, show reduced boundary separation *a* (Milosavljevic et al., 2010). When trials are manipulated so that one response is more frequent or more highly rewarded, the starting point *z* shifts to favor that response (Ratcliff and McKoon, 2008; Mulder et al., 2012; Arnold et al., 2015). When stimulus discriminability is varied, making the task harder or easier, this is reflected in changes to the drift rate *d* (Ratcliff and McKoon, 2008), while participants deprived of sleep for 24 h also show decreased drift rate (Johnson et al., 2021). Introducing a motor response handicap, such as requiring a single finger be used for all keyboard responses (Voss et al., 2004) or requiring multiple keypresses for each response (Lerche and Voss, 2019), increases *Ter*; similarly, varying the response modality (so that participants respond by eye movements, key pressing, or pointing on a touchscreen), affects *Ter* but not the other parameters (Gomez et al., 2015). Together, all these studies suggest that the DDM parameters do capture recognizable – and at least partly separable – cognitive processes.

The DDM has also been used to explore cognitive processes even when differences in observable behavior alone (e.g., participants’ response accuracy and RT) do not discriminate groups (Zhang et al., 2016). The DDM can also help disentangle different processes of information processing; for example, it has been repeatedly documented that older adults have longer RT than younger adults and that this is associated not only with higher non-decision times (*Ter*) but also with increased response caution (larger *a*; see Thiesen et al., 2021, for meta-analysis). Although originally developed to address data representing asymptotic performance on speeded response tasks where RT is fast (e.g., <1 or 1.5 s) and within-session learning is negligible (i.e., the decision rule is already known and practice effects are minimal), the DDM is increasingly also being applied to more complex tasks with longer (e.g., 1–3 s) RTs (Palada et al., 2016; Lerche and Voss, 2019; Lerche et al., 2020), to tasks that involve explicit learning across trials (Millner et al., 2018; Miletić et al., 2021), and even to data obtained from non-human animals (Brunton et al., 2013; Schriver et al., 2020).

In sum, there is a considerable and growing body of literature using the DDM to elucidate cognitive processes that affect decision-making.

## Getting started with the drift diffusion model: A concrete example

Suppose that we are going to collect data from human participants on a simple task with two stimuli (or classes of stimuli) s1 and s2 that map to two responses r1 and r2, respectively. The task has 500 trials, including 150 s1 and 350 s2 trials, so that s2 (and r2) occur more frequently than s1 (and r1). We will assume that participants could realistically achieve 100% accuracy, but the requirement to respond as quickly as possible introduces errors due to the speed-accuracy tradeoff. We might be interested in comparing two groups of participants (say, patients vs. healthy controls), and we might plan to analyze the behavioral data using one ANOVA (or non-parametric equivalent) to compare accuracy across groups, and another to compare RTs across groups. However, being aware of the speed-accuracy tradeoff, and also because we are interested in the underlying cognitive processes that produce any observed group differences, we also plan to apply a computational model, the DDM.

### Model definition

Having decided to use the DDM, the first question is: which free parameters will we consider? As noted in Table 1, DDMs usually include at least four free parameters: non-decision time *Ter*, boundary separation *a*, response bias *z*, and (at least one) drift rate *d*. Given that our task involves two different stimuli (or classes of stimuli) s1 and s2, we likely want to allow separate drift rates (*d.s1* and *d.s2*) for trials with our two types of stimuli.

Both simpler and more complicated models are possible. For example, we already noted above an example where we might want multiple drift rates corresponding to multiple task conditions. On the other hand, if we think s1 and s2 should be equally discriminable, we might assume only a single drift rate (equivalent to assuming *d.s1* = *−d.s2*: i.e., the two drift rates are of equal steepness, but slope in opposite directions, one down to r1 and the other up to r2). Our resulting model would therefore have only three free parameters that could vary across subjects: *Ter*, *a*, and a single drift rate *d*. Similarly, if we think there is no reason to assume a response bias, we might consider consider fixing *z* at 0.5. In this case, *z* would no longer be “free” to vary across participants, but would have the same value for everyone.

For now, though, let us focus on a “default” DDM with five free parameters: *Ter, a, z, d.s1,* and *d.s2*, while noting that other versions are possible. In fact, our DDM will look very much like that in Figure 3A, although different participants may have different values of the free parameters – such as the examples schematized in Figures 3B–D – that in turn produce individual differences in behavior.

At this point in the process, we would also typically consider what approach we plan to use for model-fitting, and whether software is available; for the DDM, several options exist that will be considered in more detail in the section on “Model-fitting in the DDM,” below.

### Empirical data

Continuing our example, let us assume we have a test dataset obtained from one participant on our task. (The test datafile is provided in the Supplemental material: see Appendix).

**Figure 4:**
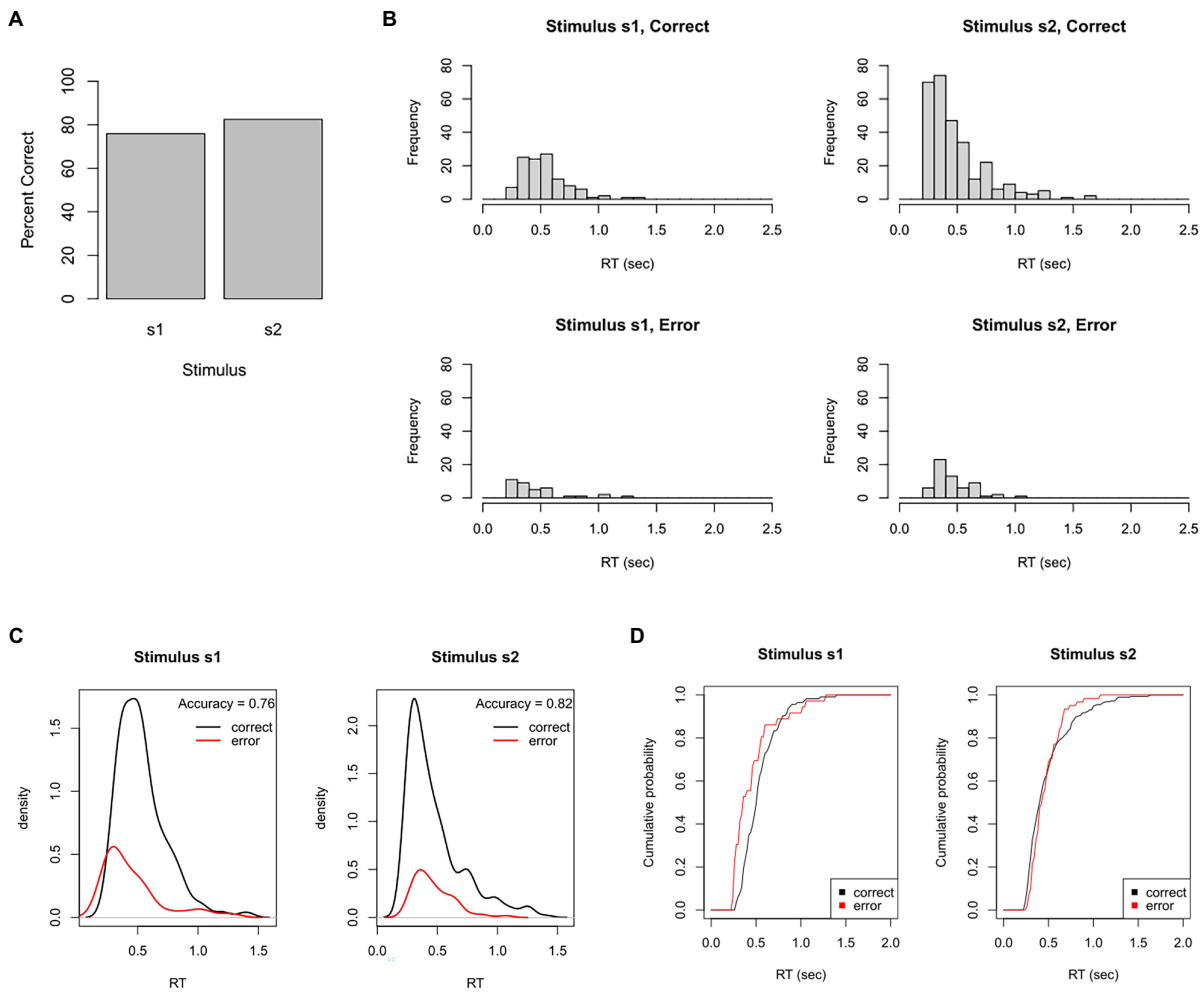
Test dataset. Here, the participant completed a task that intermixes 150 trials with stimulus s1 (correct response = r1) and 350 trials with stimulus s2 (correct response = r2). **(A)** In this example, the participant achieved 76% accuracy on s1 and 83% accuracy on s2. **(B)** Frequency histograms showing right-skewed RT distributions for each stimulus and response. **(C)** The RT data plotted as probability density functions (PDFs), in which height of the curve at any point on the x-axis reflects the probability of a response occurring at that RT. Here, PDFs are scaled so that the total area under the curves sums to one, resulting in “taller” PDFs for the more frequent correct than incorrect response. **(D)** The same data plotted as cumulative distribution functions (CDFs), showing the probability that a response has occurred at or before each RT; here, curves asymptote at about 2 s, showing that all RTs have occurred by that point. **(C)** Plotted using functions in the DMC package for R.

From these behavioral data, we could plot this participant’s overall accuracy. As shown in Figure 4A, this participant made about 76% correct responses to s1 but about 83% correct responses to the more frequent stimulus, s2. We could also plot the distribution of RTs for correct and incorrect responses to each stimulus. Figure 4B shows unimodal RT distributions with right skew: most responses occur within 250–750 msec, but a small proportion take 1 s or longer (and none occur faster than 220 msec). There also tends to be slightly faster mean RT on error responses, which is often the case when participants sacrifice accuracy for speed.

Sometimes, the RT histograms of Figure 4B are instead presented as probability density functions (PDFs), as shown in Figure 4C, in which the height at any given point on the *x*-axis corresponds to the likelihood of a response occurring at that RT. PDFs are often plotted with correct and error responses on the same graph, scaled so that the area under the curves sums to 1. This makes it easy to see not only the relative rates, but also the relative timing of correct and incorrect responses.

Figure 4D shows a slightly different way of visualizing the same data: cumulative distribution functions (CDFs) which plot at each RT the probability that a response has occurred at or before that RT. No RTs occur faster than about 0.2 s, and all RTs – for both stimuli, correct and incorrect responses – have occurred by about 1.5–2 s. (In Figure 4D, the curves are scaled to asymptote at 1, indicating that 100% of responses have occurred; sometimes, CDFs are instead scaled so that the height at asymptote represents the likelihood of each response; in this case, the curve is called a “degraded CDF.”)

While the plots in Figure 4 do convey useful information about the empirical data, they do not take into account the speed-accuracy tradeoff. In this simple task, it seems likely that our participant could have achieved 100% accuracy if instructed to work slowly and carefully – and conversely, might have made even more errors if instructed to respond even more quickly. This makes it problematic if we wished to compare a group of subjects (e.g., healthy controls) against another (e.g., neurological or psychiatric patients): perhaps one group has higher accuracy, but is this because they were more willing to sacrifice speed? And can we infer anything useful about the underlying cognitive processes in each group? For this, we turn to the DDM.

### Data cleansing

Before applying the DDM to our empirical data, we have one more step to consider: data cleansing. RT distributions are typically unimodal but right-skewed, as illustrated in Figure 4B, with a few very long RTs but no very short RTs.

It is widely assumed that genuine RTs have a theoretical minimum of about 100–150 msec, representing the time needed for the physiological processes of stimulus perception and motor response execution, and bounded by the speed of neuronal transmission in the nervous system (Luce, 1986; Whelan, 2008; Woods et al., 2015). Decision-making time (*Td*) would add (at least) tens of msec to this lower bound. However, empirical data files often contain a few “very fast” RTs of <100 msec, which could be the result of anticipatory responding initiated before stimulus onset, or even a very delayed response from a prior trial. There may also be “extremely slow” RTs, which could occur because the participant is briefly inattentive (e.g., a distracting noise in the background or a sneeze). Unfortunately, such outlier RTs can strongly influence the outcome of hypothesis tests on RT data (Ratcliff, 1993) as well as biasing any attempts at model-fitting (Ratcliff and Tuerlinckx, 2002). Therefore, it is common to perform data cleansing to attempt to reduce the effect of outlier RTs (Ratcliff, 1993; Whelan, 2008).

Various techniques for reducing the effect of outlier data have been proposed, but a common solution is to define absolute cut-points for very-short and very-long RTs, and drop from the dataset any trials with RTs outside those limits. Ideally, cutoffs should be chosen that eliminate obvious outliers while retaining as many data points as possible as possible (ideally, no more than 1%–2% of trials should be dropped). The choice of appropriate cutoff criteria for a specific study will of course vary, but common cutoffs are often RT < 100 or 200 msec and RT > 1 or 1.5 s; studies have suggested that findings may be relatively robust to minor differences in the exact cutoff values (e.g., Ratcliff et al., 2018).

Alternate methods for reducing the effect of outliers, such as transforming the data to normalize the data, or using cutoffs based on standard deviation or interquartile range, are possible, and can seem less *ad hoc*, but may greatly reduce power and can introduce biases of their own (Ratcliff, 1993; Ulrich and Miller, 1994).

In our test dataset, we inspect our data file (e.g., histograms of Figure 4B), and find no obvious outlier RTs, and we can move on.

## Model-fitting in the drift diffusion model

At this point, we are ready to use the DDM to estimate the parameter values that best describe our empirical data and – we hope – the participant’s underlying cognitive processes. The task at hand can be thought of as finding a configuration of parameter values in the DDM that, together, cause it to generate simulated data that are as close as possible to the empirical data shown in Figure 4, accounting for both accuracy rates and for the distributions of correct and incorrect RTs.

### Overview of the process

We start by proposing some “reasonable” values for the DDM parameters. For example, we might set boundary separation at an arbitrary value of *a* = 1 and starting point at *z* = 0.5 (no *a priori* bias for either response); given that RTs in the data seem to range from about 0.25–1.0 s, we might estimate non-decision time at *Ter* = 200 msec (assuming the decision time *Td* always takes at least an additional few dozen msec, so our fastest RTs would be about 220–250 msec); for drift rate, we might set *d.s1* < 0, reflecting that the evidence accumulation on s1 trials should proceed downward (toward r1), and *d.s2* > 0, so that evidence accumulation on s2 trials should proceed upward (toward r2). Here, for illustration, we’ll set *d.s1* = −1 and *d.s2* = +1.25.

To simulate a single trial with stimulus s1, we plug these parameter values into the DDM equations, start the evidence process at *z* with a boundary separation of *a* and drift rate of *d.s1*, allow the diffusion process to operate until a boundary is crossed at time *Td*, and then record both accuracy (was the correct response “r1” generated?) and overall RT (*Ter* + *Td*) for this trial. In this example, we find that the response r1 is indeed chosen, with an RT of 0.382 ms.

Since the evidence accumulation process in the DDM is noisy, we would repeat a second time, and get a slightly different RT (and potentially even a different response). In all, we repeat 500 times (150 with s1 and 350 with s2, just as in the empirical data), resulting in distributions of predicted RTs for correct and incorrect responses, and a predicted accuracy rate (how often the correct response was selected). These predictions (from the DDM) are then compared to the accuracy and RT distributions in the empirical data (from the participant) to determine how well the model fits the empirical data.

**Figure 5:**
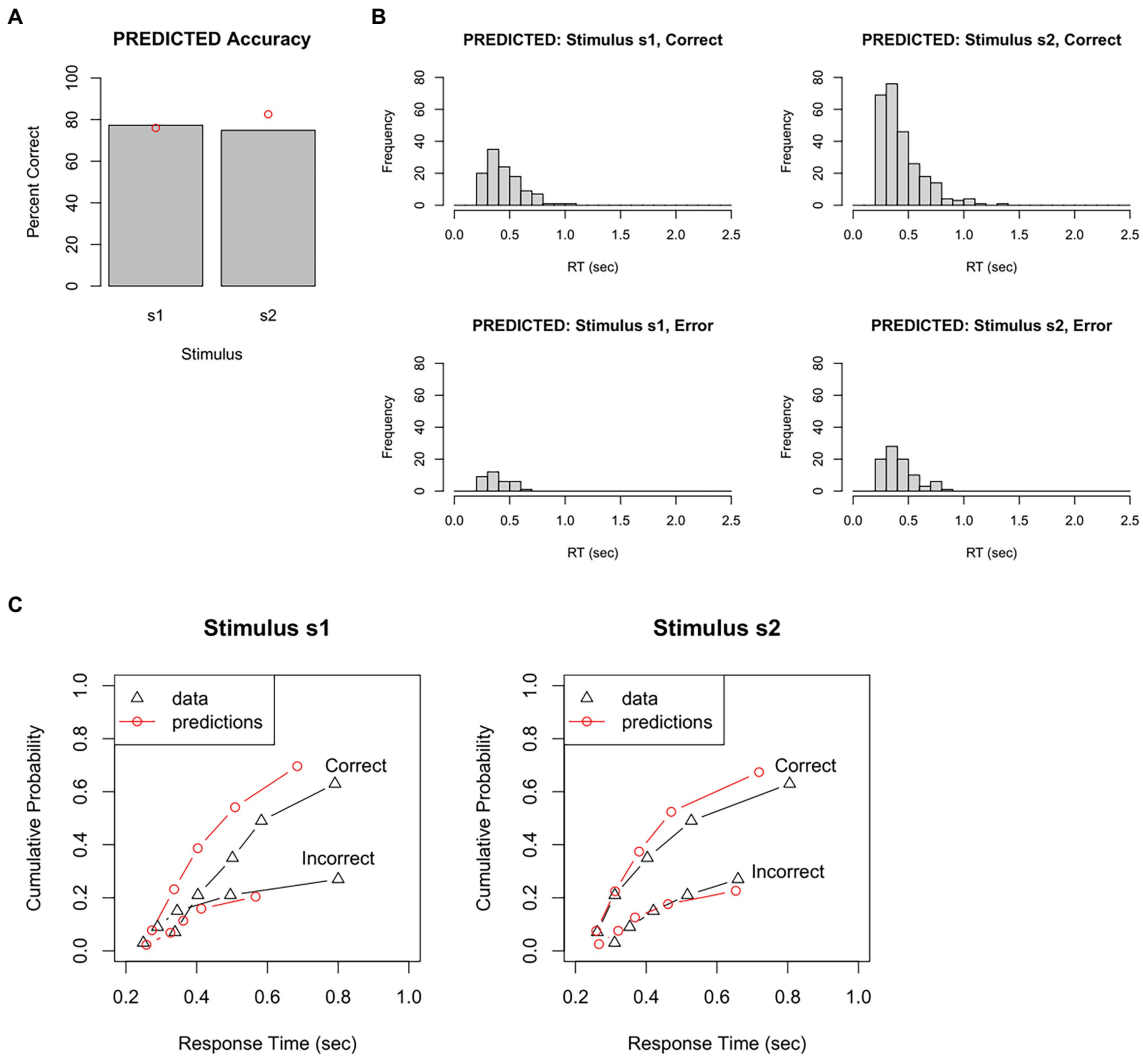
Results from a single run of the DDM, given a particular set of parameter values. On each trial, evidence accumulation occurs in the DDM until a boundary is crossed, triggering a response; the response and RT = *Td* + *Ter* are recorded for each trial. Just as in the behavioral task completed by our participant, there are 150 s1 trials and 350 s2 trials. **(A)** The predicted data has accuracy rate similar to, but not identical to, the actual accuracy in the empirical data (red dots). **(B)** The predicted data also has RT distributions similar to, but not identical to, those in the empirical data (compare Figure 4B). **(C)** One way of quantifying model fit is by dividing the empirical RT distribution into quantile “bins,” e.g., the RT at which the fastest 10, 30, 50, 70, 90, and 100% of RTs have occurred, plotted here as black triangles (for correct and incorrect responses to each stimulus). The simulated (predicted) data obtained from the DDM are divided into the same bins, plotted here as red circles, and connected by red lines to simulate a CDF. If the predicted data fit the empirical data very well, the red circles would overlie the black triangles. Here, the fit between empirical and predicted data is not great. For example, the model predicts too many correct responses and also too few slow RTs, compared to the empirical data. Predicted data obtained using the RWiener and rtdists package for R.

For example, Figure 5A plots accuracy (how often the DDM chose the correct response to each stimulus) and Figure 5B shows the distribution of RT obtained for each stimulus, for correct and incorrect responses. These predicted data can be visually compared with the empirical data (Figures 4A,B).

In this example, on s1 trials, the DDM chose the correct response (r1) 77% of the time – very close to the 76% accuracy in the empirical data. On s2 trials, the DDM chose the correct response (r2) 75% of the time – a little lower than the 83% accuracy in the empirical data. The histograms for the predicted data also look similar to those from the empirical data, although not perfect: for example, the mean for correct responses to s1 is about 0.44 s, which is a little faster than the mean 0.54 s in the empirical data.

We could also plot PDFs and CDFs of the predicted data, to see how closely these overlap the PDFs and CDFs from the empirical data. For example, the empirical data could be divided into quantile “bins,” for example, based on the RT at which the fastest 10%, 30%, 50%, 70%, 90%, and 100% of RTs have occurred. For our test dataset, this would result in an approximation to a degraded CDF shown by the black lines in Figure 5C. The simulated data generated by the DDM can be plotted in the same way (red lines in Figure 5C). In this example, the correspondence between empirical and predicted data is not great. For example, according to Figure 5C-left, 90% of incorrect responses to stimulus s1 have occurred by about 800 msec; the model, however, predicts that 90% of error responses to s1 will have occurred by about 600 msec. In other words, the particular set of DDM parameter values used to generate these predicted data do not reproduce the empirical RT data very accurately.

All of the above has allowed us to assess how well the DDM reproduces a single participant’s data, given a single set of parameter values in the model. We could then slightly perturb one or more of our parameter values – say, changing *a* from 1 to 1.01 – and run the DDM again to see if the new combination of parameter values provided a better approximation to the participant’s data. After iterating over a large number of possible parameter values, we would obtain the best possible fit: a set of values for *a*, *z, Ter* and drift rates *d.s1* and *d.s2* that together allow the DDM to most closely replicate the empirical data.

Needless to say, estimating the optimal values for multiple free parameters is a formidable computational challenge, one that is complicated by the fact that changes to one parameter may affect the optimal value of another parameter. Fortunately, several methods have been devised, many of which are currently available as open-access software packages.

### Specific methods and computational packages for parameter estimation using drift diffusion model

This section reviews several methods of parameter estimation for DDM that have been widely used in the literature, and that have been implemented as freely-available software.

#### *χ*^2^ method

An early, and mathematically tractable, approach to estimating DDM parameters is the *χ*^2^ method (e.g., Ratcliff and Tuerlinckx, 2002), which compares a histogram of RT distributions in the empirical data to those predicted from the model under a given set of parameter values (as in Figure 5C). For those familiar with the *χ*^2^-test in inferential statistics, that method distributes the empirical data into a number of bins or categories, and then compares that distribution against the distribution predicted by the null hypothesis; the *χ*^2^ statistic is a measure quantifying the difference in distributions, and if *χ*^2^ is large enough, the null hypothesis of no difference between distributions can be rejected. Conversely, if the distributions are similar (i.e., small *χ*^2^), then the model is said to provide a good fit to the empirical data.

The goal is to identify a set of DDM parameter values that, together, minimize *χ*^2^ – minimizing the difference between empirical data and DDM predictions. Without delving too deeply into the mathematical methods, suffice to say that this can be done *via* an optimization routine. Many optimization routines involve iterative search: start with an initial rough estimate of the parameter values, calculate how well the model using those values fits the data, and then iteratively perturb one or more of the values, to see if this provides a better fit: if yes, then the new values are adopted for the next pass; if no, then the old values are retained and a different perturbation is tried. Early in the search, when the fit is likely to be poor, minor perturbations of parameter values may produce large improvements in fit; but the search is said to converge when values for all parameters have stabilized and no further perturbations can be identified that improve fit by more than a very small value (e.g., 10^−7^). At this point, the value of *χ*^2^ is returned as a metric of goodness-of-fit, and the corresponding parameter values are taken as the optimal or “best-fitting” parameter estimates for the empirical data.

The *χ*^2^ method is one of the estimation methods instantiated in the freely available fast-dm package (Voss and Voss, 2007; Voss et al., 2015) and it can also be used with the rtdists package in R (Singmann et al., 2016).

The main advantages of the *χ*^2^ approach are computational speed and relative robustness to outlier RTs. The robustness to outliers reflects the fact that the first and last bins are “open” in the sense that there is no lower bound for RT in the quantile bin that contains the fastest responses, and no upper bound for RT in the bin that contains the slowest responses; therefore even a very extreme outlier (say, RT = 0 s or RT = 10,000 s) would not dramatically affect the results. However, the *χ*^2^ approach requires a large number of trials to produce a reliable estimate (e.g., at least 500 trials) and may be especially problematic if there are relatively few error responses (e.g., <12 trials in any quantile bin; Voss et al., 2015).

For these reasons, the *χ*^2^ approach to parameter fitting has become less widely used in recent years, as other methods have become available, and as computing power has increased. However, it’s worth understanding this method because RT quantiles (such as those in Figure 5C) are often plotted in publications that have used other model-fitting methods.

#### Maximum likelihood estimation

A popular method for estimating DDM parameters uses maximum likelihood estimation (MLE) to generate estimates for each parameter. MLE may be most familiar to readers as a means to identify parameter values (beta weights) in regression models, to minimize difference (error) between the predicted and empirical outcomes. The principle is the same here.

Formally, MLE tries to find a set of parameter values that, together, maximize the probability that the outcome of the model matches the empirical data on all trials. This probability is referred to as the likelihood estimate, *L*; more commonly, researchers report the log of that value, which is called the log-likelihood estimate *LLE*.

The goal then becomes to find the set of parameter values that, together, maximize *LLE* for the model applied to a given dataset. Again, this is normally done by optimization routines that work by iterative search: constructing a (possibly random) set of “starting values” for the parameters and evaluating *LLE*, then perturbing one or more parameters by a small amount and re-evaluating *LLE*, until no further improvements in *LLE* can be obtained by perturbing any of the parameters. (Some researchers prefer to speak in terms of *minimizing* negative *LLE*, rather than *maximizing* positive *LLE*, but the resulting parameter estimates will be the same.)

**Figure 6:**
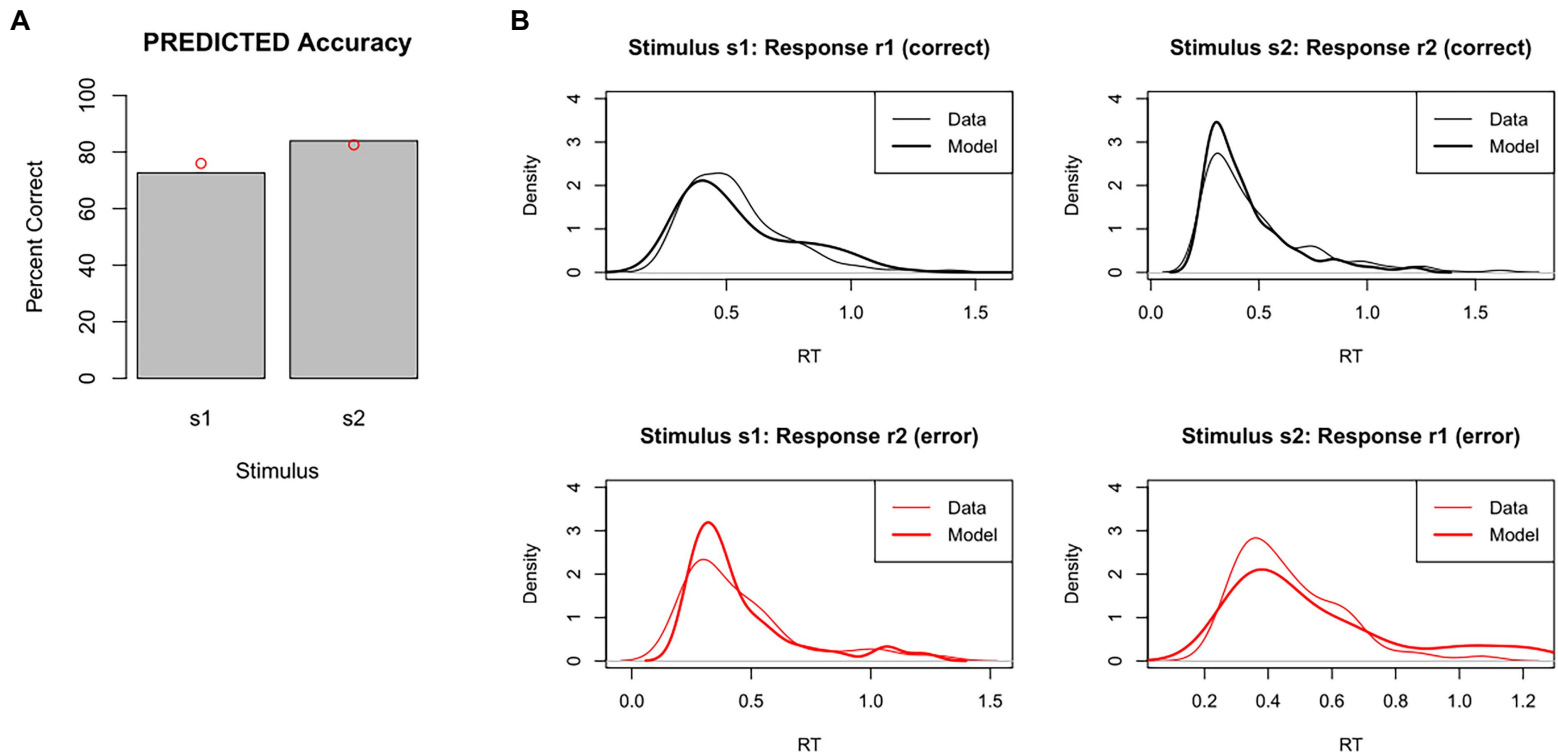
Results of model-fitting using maximum likelihood estimation (MLE). The set of DDM parameter values is identified that, together, maximizes the likelihood of the model producing same response as the participant on each trial. Given these “best-fitting” parameters, **(A)** accuracy levels for s1 and s2 in the predicted data were quite similar to those of the empirical data (red dots), and **(B)** RT distributions for each combination of stimulus and response were also quite similar in predicted and empirical data. DDM results obtained using RWiener package in R.

For example, Figure 6 shows accuracy and RT distributions obtained after using MLE to optimize the model parameters against our test dataset. The figures show that the predicted data matches the overall accuracy of the empirical data pretty well, although predicting slightly too few correct responses to s1 and slightly too many correct responses to s2, and also captures the modes and general shape of the RT distributions reasonably well.

Freely-available software implementing MLE for DDM includes the fast-dm package (Voss and Voss, 2007; Voss et al., 2015) as well as the RWiener (Wabersich and Vandekerckhove, 2014) and rtdists (Singmann et al., 2016) packages for R.

MLE approaches have been successfully used in a large number of DDM studies, and can be used even when there are relatively few (e.g., <50) trials available from each participant (Lerche et al., 2017); however, MLE can be very sensitive to outlier RTs (especially very fast RTs), and so careful thought must be given to data cleansing. MLE algorithms are also vulnerable to local minima, meaning that they can converge to a set of parameter values where no small perturbations can further improve *LLE*, even though this may not be the optimal solution. For this reason, it’s often a good idea to run the MLE procedure multiple times, with different starting values, to make sure the same solution is found each time.

#### Bayesian approaches

Recently, the advent of open-source platforms for Bayesian statistics have given rise to a number of Bayesian approaches for estimating DDM parameter values (for readable introductions to Bayesian methods, see Kruschke and Liddell, 2018; Wagenmakers et al., 2018b). In brief, these methods follow a Bayesian approach starting with initial estimates (i.e., “prior distributions” or simply “priors”) about reasonable values for each parameter that are iteratively updated to produce “posterior distributions” (or simply “posteriors”) for those parameters.

**Figure 7:**
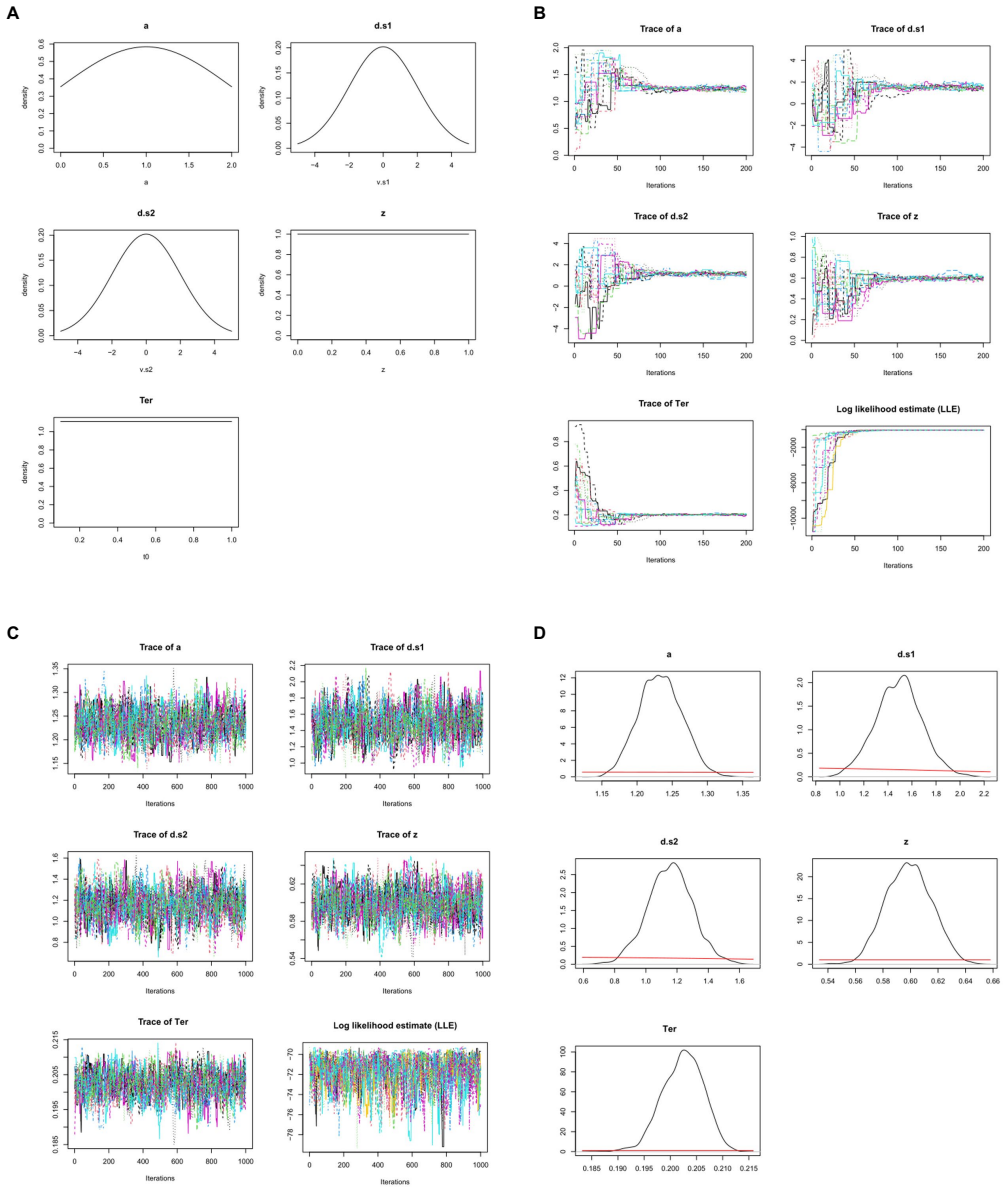
Bayesian approaches to estimating DDM model parameters. **(A)** First, prior distributions or “priors” (initial estimates) are generated for each model parameter. Here, the prior for non-decision time *Ter* simply specifies that it is a value in the range from 0.1 to 1.0 s, with all values in that range equally likely (uniform distribution); similarly, the prior for starting point *z* is a value in the range from 0 to 1.0 (as a proportion of boundary separation, a). The priors for drift rates are specified as broad normal distributions with means of 0; the prior for a is a normal distribution with mean of 1, truncated at 0 and 2 (because boundary separation is in arbitrary units but can never be <0). These priors are all intentionally vague, so that they have minimal influence on the posteriors. Note that, in these graphs, drift rates *d.s1* and *d.s2* are plotted as >0 if they slope in the direction of the correct response. **(B)** MCMC is applied to the dataset from Figure 4, using priors as defined in **(A)**: 15 “chains” are formed starting from the prior distributions and progressively updating, with model fit evaluated at each step. Traceplots of values for the 5 DDM parameters over the first 200 iterations are shown as one colored line per chain. Initially, there is wide variation across chains in the initial values drawn for each parameter from the priors; however, over the first 200 iterations, parameter values start to converge to similar values in all chains. For example, *Ter* (which the priors merely specify as ranging between 0.1–1.0 s) quickly converges to a mean value near 0.2 s. As the parameters begin to converge, log-likelihood estimates (LLE) for each chain also improve rapidly, indicating improving model fit. **(C)** Following the initial 200 trials (which are discarded as “burn-in”), an additional 1,000 iterations are run; the traceplots for each parameter now resemble “fat, flat, hairy caterpillars”: relatively horizontal with small, random scatter around a common mean value, which is a visual indicator that estimates for the parameters have converged. Meanwhile, LLE for the chains is also relatively stable within and across chains: compare scale of *y*-axes in **(C)** vs. **(B)**. **(D)** The resulting posterior distributions or “posteriors” for each parameter. The mean/median of posteriors can be taken as a point estimate of the parameter value, and the width is a measure of uncertainty in that estimate. Compared to the priors, posteriors should be unimodal and fairly narrow: Note the difference in x-axis and y-axis scales from **(A)** to **(D)**. For visual comparison, the priors are plotted in **(D)** as red lines, and at this scale they appear as nearly flat lines near *y* = 0. For example, whereas the prior estimate for *z* treated all values in the range from 0 to 1 as equally plausible, the posterior estimate for *z* has narrowed to a distribution with mean 0.60 and SD 0.02, indicating a mild but definite response bias (toward the upper boundary and r2). DDM results and figures from DMC package for R; all plots are arbitrary units except *Ter*, which is in sec.

Often, very vague and uninformative priors are used, so that minimal pre-existing knowledge is assumed and even a small amount of data will “overwhelm” the priors, meaning that the posteriors depend much more on the data than on the researcher’s choice of priors; additionally, several different priors may be tried, to show that the prior assumptions do not greatly influence the posteriors/conclusions. For example, the prior for *z* might simply specify that it is a value somewhere in the range from 0.0 to 1; the priors for drift rate might specify broad normal distributions with mean 0; etc. (see Figure 7A).

Whereas the *χ*^2^ and MLE methods attempt to estimate a single “best-fitting” value for each parameter, Bayesian approaches generate posterior distributions for each parameter, including both a point estimate (e.g., mean or median) and a measure of confidence in that estimate (e.g., the standard deviation SD or confidence interval CI). For example, Figure 7B shows posterior distributions (or simply “posteriors”) for the five DDM parameters; for visual comparison, the priors are plotted as red lines, and at this scale they now appear as nearly flat lines near *y* = 0. For example, whereas the prior estimate for *z* treated all values in the range from 0 to 1 as equally plausible, the posterior estimate for *z* has mean 0.60, indicating a mild but definite response bias (toward the upper boundary and r2); the narrow width (SD = 0.2) indicates high confidence that the true value lies in the body of the posterior. In this case, we can say that information from the data has “overwhelmed” the priors, resulting in much more closely specified posteriors.

Calculating posterior distributions is extremely computationally intensive, and direct solution is generally intractable: i.e., there is no known mathematical way to directly calculate the posteriors from the priors and the data. Instead, approaches such as Markov Chain Monte Carlo (MCMC) methods leverage computer power to generate approximate solutions (for a readable introduction to MCMC methods, see van Ravenzwaaij et al., 2018).

In brief, MCMC methods estimate a distribution by repeatedly drawing “sample values” to form a “chain” of values for each parameter. A simple version of MCMC might run as follows: At the first step or iteration of the chain, a value is selected at random for each parameter from the prior distributions, and the resulting model is evaluated (here, the DDM would be run and *LLE* computed). At the next iteration, the distribution of one of the parameters is perturbed slightly, perhaps by slightly altering its mean or SD, and new parameter values are drawn at random from the distributions, and the model is run again. If the result is an improvement (e.g., improved *LLE*), then the updated parameter values are used for the next step in the chain; otherwise, the old parameter values are retained. The process is repeated hundreds or thousands of times, until no further improvements are discovered, at which point the process is said to have converged on a solution: a set of distributions (posteriors) for each parameter.

If we are examining a DDM with five free parameters (*a, z, Ter, d.s1, d.s2*), then typically all the parameter values are updated at each iteration (often, by holding all the other parameters constant at their current values while we perturb and evaluate each one in turn). Therefore, each sample in the chain contains updated values for all the parameters.

Typically, multiple chains are run, often using the rule of thumb to run three times as many chains as there are free parameters; thus, for a DDM with five free parameters we may run 15 chains. Results from 15 such chains are shown in Figure 7B, one colored line per chain. The figure shows that, at the first iteration, there is a wide variety parameter values drawn from the priors; but within a few dozen iterations, the parameter values in all chains begin to converge to common values, and *LLE* rapidly increases for all the chains.

For example, the prior for *Ter* is a uniform distribution in the range from 0.1–1.0 s (refer Figure 7A). At the start of each chain, a value for *Ter* is chosen at random from that distribution; because the prior is vague, starting points can vary widely, as shown at the extreme left of the *Ter* traceplot in Figure 7B. Across 200 iterations, though, all the chains gradually converge to new estimates of *Ter* with means close to about 0.2 s. This rapid fine-tuning of *Ter* and the other parameters produces corresponding improvement in *LLE*, reflecting progressively better model fit. Here, by about the 200th iteration, all the chains are meandering around the same point, without any large deviations or upward or downward trends.

Because of the wide variability in possible starting points, the beginning of each chain (e.g., the first 200 iterations shown in Figure 7B) is often discarded (as “burn-in”), and then the chains run for a few more hundreds (or thousands) of iterations, so that the posterior predictions are based on the (hopefully stable) ends of the chains, as shown in Figure 7C. Note the change in scale on the y-axes from Figures 7B,C as the chains “zero in on” a very narrow distribution of values for each parameter, resulting in only minor fluctuations in *LLE* from one iteration to the next.

Formally, the state when the distribution does not change (much) across iterations is known as convergence. Convergence can be visually assessed by inspecting the traceplots of the chains, which should look like the “fat, flat, hairy caterpillars” of Figure 7C: minor fluctuations around a mean value with no systematic upward or downward tendencies (compare the unconverged chains in the early iterations of Figure 7B).

Convergence can also be assessed quantitatively. One widely-used measure of convergence is the Gelman-Rubin Rˆ (“R-hat”) statistic (Gelman and Rubin, 1992) which assesses the similarity of within-chain vs. between-chain variability. Values of Rˆ approaching 1 indicate convergence; a common criterion for good convergence is Rˆ < 1.1. (In the example from Figure 7, after 1,000 iterations, Rˆ = 1.03.)

Given successful convergence, the posterior distributions can be reported (e.g., Figure 7D), and/or the mean or median of the posteriors can be used as point estimates of the parameters.

Freely-available software implementing Bayesian approaches to DDM includes the python-based HDDM (Wiecki et al., 2013) and the Dynamic Models of Choice (DMC) package for R (Heathcote et al., 2019).

Bayesian approaches to DDM may be more robust in recovering model parameters than other methods, such as MLE and *χ*^2^ methods, when a limited numbers of trials are available (Wiecki et al., 2013). Bayesian approaches also provide not only parameter estimates (mean or median of the posterior distributions), but also quantify the uncertainty in those estimates (standard deviation or 95% confidence interval of the posterior distributions). Like MLE algorithms, Bayesian methods based on iterative sampling may be vulnerable to getting stuck in local minima, although this risk is ameliorated by use of multiple chains to ensure convergence (e.g., the “hairy caterpillars” of Figure 7C indicate that all the chains are converging around the same stable estimates).

#### Hierarchical methods

The above methods for estimating DDM parameters all assume that parameters are fit to each participant’s data independently. An alternate approach is hierarchical modeling, which addresses individual differences while also pooling information across individuals to generate group-level parameter estimates (Vandekerckhove et al., 2011; Wiecki et al., 2013; Johnson et al., 2017). Hierarchical approaches may be particularly useful where within-group variability is much lower than between-group variability, or where only a small number of trials are available for each participant; however, hierarchical models may not be valid if there are only a few participants in each group. The Bayesian model approaches in some software packages, including HDDM and DMC, provide for hierarchical model fitting.

### So, which method should be used?

Each of these methods for estimating parameters has strength and weaknesses. Table 2 summarizes a few of the key considerations when determining whether to use *χ*^2^, MLE, or Bayesian methods. Choice of an appropriate method for a given dataset typically represents a compromise among these considerations, as well as the researchers’ familiarity with a particular approach and access to software. Fortunately, the availability of well-documented software packages, and widespread availability of powerful computers, means that all these methods are within the reach of an investigator willing to invest the time required to learn their use.

**Table 2:** Comparison of some key features among three approaches to estimate DDM parameters.

	*χ*^2^ method	Maximum likelihood estimation (MLE)	Bayesian approaches (based on MCMC sampling)
Computational tractability	Relatively fast	Moderate	Can be very slow
Sample size required	Requires large number of trials per subject (e.g., at least 500 trials with at least 12+ per quantile bin)	Can be used with as few as about 40–50 trials per subject, at least 10 trials per condition	May be more robust than other methods when limited number of trials available
Outlier RTs	Relatively robust to outlier RTs	Very sensitive to outlier RTs (especially fast RTs)	Moderately sensitive to outlier RTs
Other (+ and −) considerations	− Loss of information due to use of quantile bins, rather full RT distribution	+ General principles of MLE are likely familiar to a wide swath of researchers	+ Allows to quantify not only parameter estimates, but uncertainty (variability) in those estimates
			+ Available methods for hierarchical model-fitting
			− Steep learning curve for researchers not familiar with Bayesian methods

In most cases, an investigator with access to reasonable computer power is likely to choose between MLE and Bayesian approaches. These approaches do not require the large number of trials required by the *χ*^2^ method, although they are more vulnerable to outlier RTs. For this reason, data cleansing is important to mitigate the effects of outliers.

In general, MLE and Bayesian approaches should return comparable results, although parameter estimates may differ both due to randomness (noise) in the estimation routines and also due to scaling factors adopted by different software packages.

For example, both MLE (*via* the RWiener package in R) and Bayesian MCMC (*via* the DMC package in R) were used to estimate the five DDM parameters for our test dataset. As shown in Table 3, both methods return nearly identical estimated values parameters for *a, z* and *Ter*; estimated values of drift rate differ slightly, but the relative relationships are preserved: *d.s1* is in the opposite direction to, and steeper than, *d.s2* in both methods.

**Table 3:** Parameter estimates for DDM with five free parameters, applied to the dataset of Figure 1, using MLE and Bayesian MCMC (means of posterior estimates are shown), and “true” generating parameters.

Median estimated parameter values					
	*a*	*d.s1*	*d.s2*	*z*	*Ter*
MLE	1.17	−1.35	0.94	0.6	0.201
Bayesian MCMC	1.23	−1.49	1.16	0.6	0.202
True (generating) values	1.2	−1.25	1.1	0.6	0.2

MLE, maximum likelihood estimate (calculated via RWiener package in R); MCMC, Markov Chain Monte Carlo (calculated via DMC package in R); *Ter* given in seconds; other DDM parameters (*a*, *d.s1*, *d.s2*, *z*) given in arbitrary values.

The bottom line is that, in many cases, the general conclusions from the DDM should be roughly the same, regardless of which specific approach (or software package) is used to generate parameter estimates. For many purposes, the choice will simply reflect the approach and/or software package with which the investigator is most comfortable.

Now it’s time for a big reveal: the original “empirical” test dataset (shown in Figure 4) was itself generated from a DDM with predefined parameters: *a* = 1.2, *d.s1* = −1.25 (sloping down toward the lower boundary), *d.s2* = +1.1 (sloping toward the upper boundary), *z* = 0.6, and *Ter* = 0.2. That means that we have the luxury of knowing the correct answer: if the DDM is functioning as advertised – if it is truly able to “infer” parameter values by inspecting the data generated – then the parameter estimates should be very close to the true (or “generating”) parameter values. In fact, Table 3 shows that both instantiations of the DDM do, indeed produce parameter estimates that are very close to the generating parameters.

Of course, if this dataset had been generated by a human being, we would not have the luxury of knowing the generating parameters – the entire point of using the DDM would be to attempt to infer these latent parameter values from the empirical data. But the ability of the DDM to accurately recover parameters from a test dataset greatly increases our confidence that that the methods can be applied to this type of data.

### Running the drift diffusion model on a group of empirical data files

All the work we have done so far has rested on attempting to fit the DDM to a single data file, and a “simulated” data file at that. Now let us assume we were going to fit the model to a group of data files obtained from multiple participants an experiment. For the purposes of example, let us consider *n* = 10 empirical data files obtained from our simple task (included in the Supplemental material; see Appendix). We will also assume that we have already performed a data cleansing step, to identify any trials with very-short or very-long RT (none were identified). We then run our DDM (with five free parameters *a, z, d.s1, d.s2, Ter*) using MLE (*via* the RWiener package in R) to find best-fitting parameters for each data file. The results are shown in Table 4, along with the maximum *LLE* obtained for each data file, given those best-fitting parameters.

**Table 4:** Parameter estimates obtained from the DDM, using MLE to maximize LLE for each data file separately.

	Estimated parameters					LLE
	*a*	*z*	*d.s1*	*d.s2*	*Ter*	
1	1.09	0.46	−3.93	1.27	0.21	185.2
2	1.91	0.48	−3.66	1.97	0.25	70
3	1.85	0.51	−5.3	1.8	0.16	128.5
4	1.19	0.55	−3.62	2.09	0.17	287.1
5	1.1	0.46	−4.01	1.27	0.21	189.7
6	1.65	0.49	−2.41	2.43	0.25	140.5
7	1.2	0.44	−2.01	2.02	0.21	129.5
8	0.78	0.4	−1.01	1	0.23	220.4
9	0.83	0.53	−3.2	1.53	0.18	331.9
10	0.85	0.43	−1.2	0.11	0.18	135.6
Mean	1.25	0.48	−3.04	1.55	0.2	181.8
SD	0.42	0.05	1.36	0.67	0.03	79.6

Parameter estimates obtained using RWiener package in R.

## Validating the model

The estimated parameters in Table 4 represent the configuration of parameter values (*a, z, d.s1, d.s2* and *Ter*) that, together, allow the DDM to most closely approximate each individual participant’s behavior on the task. At this point, there are a few ways in which we should validate our model, bolstering confidence that the DDM is actually discovering parameter values that describe the underlying processes that generated the data.

### Sanity check

Before going any further, the importance of a simple sanity check cannot be overstated. Do the model results even make sense?

One type of sanity check has already been mentioned: in the context of Bayesian MCMC methods, researchers often report Rˆ and/or show “hairy caterpillars” to document convergence (e.g., Figure 7C), and may plot posterior distributions to show that the parameter estimates are unimodal and (ideally) low in variance (e.g., Figure 7D). For MLE methods, optimization routines also usually report whether a predefined criterion for convergence has been met for each file.

If the parameter estimation process did not converge, then obviously the parameter estimates cannot be trusted. Sometimes, failure to converge just indicates an unlucky choice of starting point, and if the optimization routine is re-run with a different randomly selected starting point, convergence may be achieved. In this case, it’s customary for authors simply to note how many attempts (re-starts) were required before convergence was achieved.

Assuming convergence, the next step should always be a sanity check of the parameter estimates obtained. For example, non-decision time *Ter* cannot be lower than the empirically-observed behavioral RT (since RT = *Ter* + *Td* and *Td* cannot be less than zero), and boundary separation *a* is in arbitrary units but cannot be <0 (since it represents a distance or separation). In Table 4, the values of *a, z* and *Ter* all meet these minimal criteria.

If there is more than one drift rate, sign (direction) and steepness (slope) should be consistent with observed accuracy and relative response speeds. In Table 4, for all subjects, *d.s1* < 0 and *d.s2* > 0, meaning that in each case the drift rate slopes toward the correct boundary. The magnitude (absolute value) of the drift rates suggests that the evidence accumulation process on s1 trials is somewhat steeper than on s2 trials. This might reflect something about the underlying nature of the task (e.g., perhaps it is harder to distinguish and decide to respond to s2 stimuli than s1 stimuli). In any case, the drift rates in Table 4 look reasonable too.

In sum, then, our parameter estimates all appear plausible. In general, any parameter values that violate common sense likely indicate that the model has failed, regardless of what fit metrics may be reported.

### Predictive check

A next important step to establish model validity is a predictive check, in which the parameter estimates obtained from the empirical data are used to generate simulated datasets. Specifically, a DDM with the estimated parameter values is run on the same task (same number and type of trials as in the original task) and the model’s predicted response and RT recorded for each trial. This could be done one or more times for each data file, or for a few representative data files selected at random, or even using the group means for the parameter estimates.

The simulated data should mimic key features of the behavioral data, such as accuracy and mean/SD of RTs for correct and incorrect responses to each stimulus. PDFs (or RT histograms) and CDFs for simulated data can also be visually compared against the empirical data (similar to comparison of predicted vs. empirical data in Figure 6).

**Figure 8:**
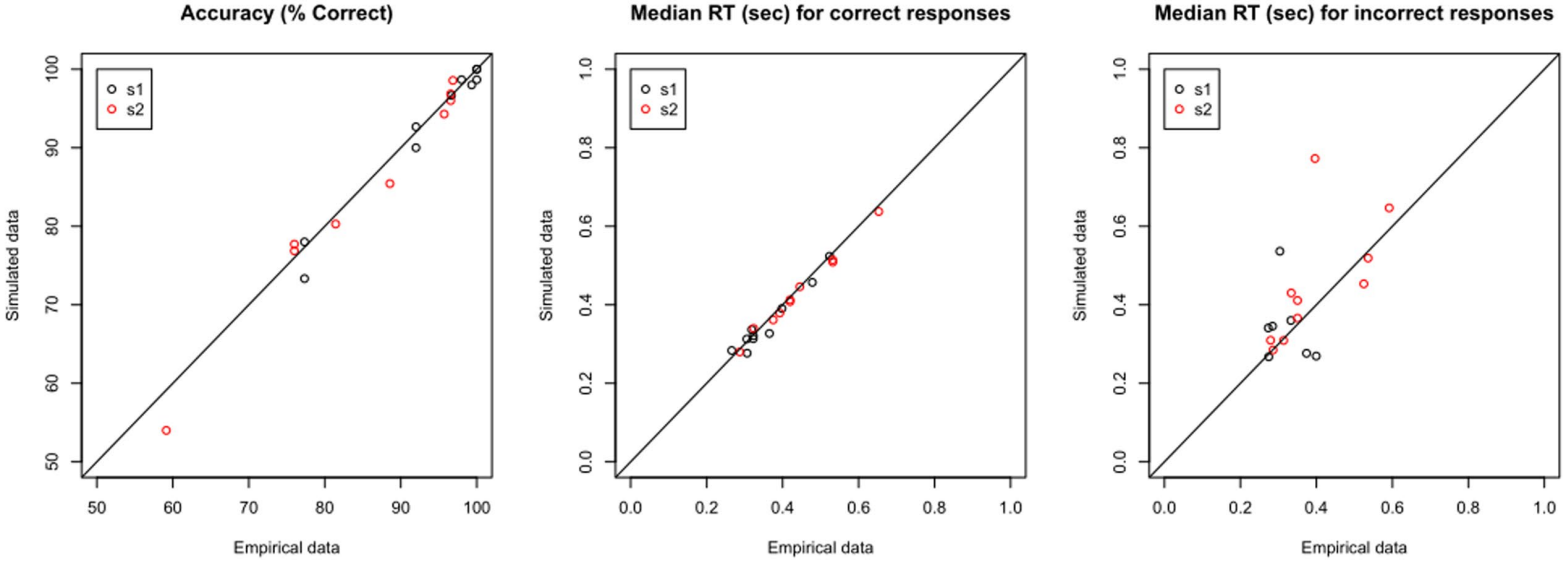
Validating the model: Predictive Check. Using the estimated parameters for each data file, shown in Table 4, the DDM is used to generate “simulated data.” For each empirical file, the simulated data should closely reproduce (left) accuracy as well as features of the RT distribution, such as median RT on (center) correct and (right) incorrect responses.

If the model predicts the empirical data well, the plots for simulated and empirical data for each participant will be highly overlapping. If we have a large dataset, rather than inspect each simulated data file individually, it may be enough to “spot-check” a few representative cases, and then look at some summary statistics, such as mean percent accuracy and median RT in the simulated vs. empirical data. Yet another possibility is shown in Figure 8: For each data file, there should be very close correspondence between the percent accuracy to s1 and to s2 in the empirical vs. simulated data, and similarly the median RT for correct and incorrect responses in the empirical data should be closely matched in the simulated data. In the current example, Figure 8 shows extremely high correlation between empirical and simulated data on percent accuracy and median RT on correct responses (all *r* > 0.97); for RT on incorrect responses, the correlation is lower, particularly for s1, reflecting the relatively low number of error responses on which these calculations are based.

The fact that the simulated data share many of the features with the empirical data cannot, of course, *prove* that they were generated in the same way – but failure would almost certainly argue that the model is *not* valid. So, predictive checks are a conventional first step in model validation.

### Parameter recovery study

After generating the simulated data, a parameter recovery study can be conducted, in which the DDM is applied to the simulated data, to see whether the parameter values which generated those simulated data can be correctly recovered by the DDM (e.g., Ratcliff and Childers, 2015; Lerche et al., 2017; White et al., 2018b).

**Figure 9:**
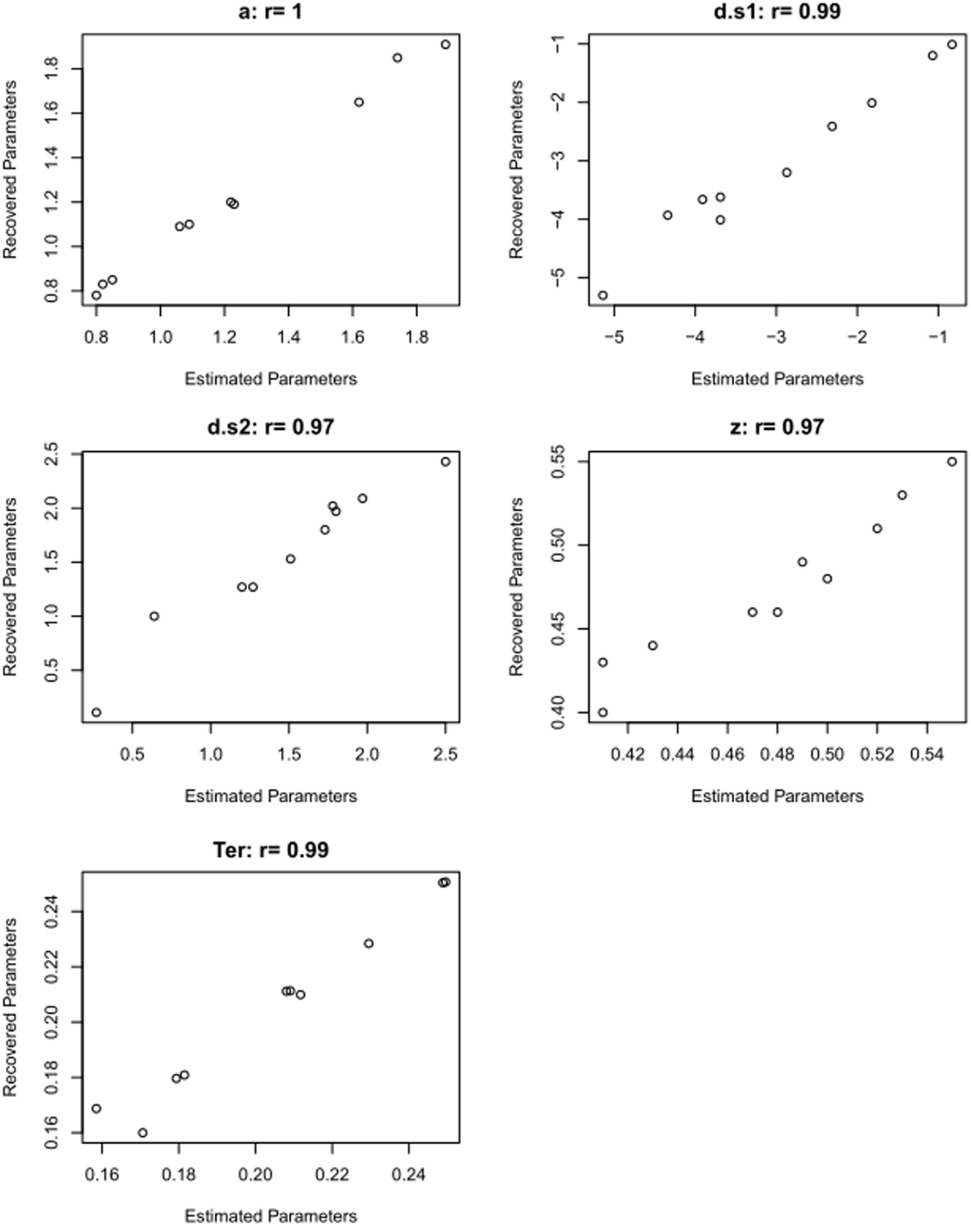
Validating the model: results from parameter recovery study. Scatterplots showing correspondence between estimated parameters (Table 4) and recovered parameters (Table 5), for the 10 simulated data files. Correlations of estimated and recovered values are excellent (Pearson’s *r* > 0.9 for all parameters). This increases our confidence that the DDM can accurately recover parameter values for this type of data.

Table 5 shows the results of just such a parameter recovery study: using the estimated parameters from each participant (Table 4) to generate 10 simulated datasets, and then running the DDM on those simulated datasets to infer or “recover” those parameter values. In a perfect world, the parameter values estimated from the simulated data will match the generating parameters quite closely: high correlation (Pearson’s *r*) between generating and recovered parameters is considered “good” if *r* > 0.75 or “excellent” if *r* > 0.90 (White et al., 2018b). For the current example, as shown in Figure 9, the correlations between generating and recovered parameters would all be considered excellent (all Pearson’s *r* > 0.9).

**Table 5:** Parameter recovery test: estimated parameters “recovered” from simulated data.

	Recovered parameters					*LLE*
	*a*	*z*	*d.s1*	*d.s2*	*Ter*	
1	1.06	0.48	−4.34	1.2	0.21	198.8
2	1.89	0.5	−3.91	1.8	0.25	53.2
3	1.74	0.52	−5.14	1.73	0.17	129
4	1.23	0.55	−3.69	1.97	0.16	264.5
5	1.09	0.47	−3.69	1.27	0.21	156.8
6	1.62	0.49	−2.31	2.5	0.25	144.8
7	1.22	0.43	−1.82	1.78	0.21	57.6
8	0.8	0.41	−0.83	0.64	0.23	180.3
9	0.82	0.53	−2.87	1.51	0.18	334.1
10	0.85	0.41	−1.07	0.27	0.18	127
Mean	1.23	0.48	−2.97	1.47	0.2	164.6
SD	0.39	0.05	1.43	0.65	0.03	86.3

Parameter estimates obtained using RWiener package in R.

A successful parameter recovery study confirms that the model-fitting procedure is able to reliably estimate (“recover”) the parameter values that generated the simulated data. This cannot, of course, guarantee that the model has accurately estimated parameter values from the empirical data, but it does increase our confidence that the model is (at least) *capable* of correctly recovering parameters given data such as these. If the model cannot even replicate the generating parameters for simulated data, where we know what the true values for each parameter are, then we certainly cannot trust that it is accurately estimating the parameters for human participants, where the generating parameters are not known!

## Model selection

The previous section focused on how we find a set of estimated parameter values that provide the “best possible” fit for each file in our empirical data. It’s also important to ask just how good that “best possible” fit actually is. Model selection typically refers to a process of systematically comparing different instantiations (or versions) of that model, with different free parameters, to determine which provides the best way of understanding the data.

For example, in the preceding sections, we used a DDM with five free parameters, including *a, z, Ter* and separate drift rates *d.s1* and *d.s2* for s1 and s2 trials, and we obtained pretty good results, validated both by predictive check and parameter recovery study; but could we have obtained (almost) as good results with a simpler model, say, assuming only a single drift rate *d*, regardless of stimulus?

Other things being equal, we would typically favor the simpler model with fewer free parameters, as a simpler way to describe the data, and also because models with more free parameters have a higher risk of overfitting the data. Overfitting refers to situations where a model can describe an existing dataset with high accuracy, but does not generalize well to other datasets. This is a concern not only in the DDM, but in all kinds of model fitting, such as linear regression: adding a large number of predictor variables to make a more complex model may result in overfitting the sample data, such that the regression equation obtained makes very accurate predictions on the sample data, but not for new datasets. A simpler regression model, with fewer predictor variables may sacrifice some accuracy but provide better generalization.

So, do we really need two separate drift rates in our model? Are the benefits (better model fit) worth the costs (complexity and potential overfitting)? To answer this question, we need to evaluate two things: first, exactly how “good” a fit does each version of the model provide to the data? Second, is one fit “meaningfully” better than the other?

### Assessing model goodness-of-fit

In order to quantify model fit, several goodness-of-fit metrics are available. We have already discussed one: the maximal *LLE*. The maximal *LLE* is simply the value of *LLE* that was obtained using the best-fitting parameter values (and it was reported for our example study in Table 4).

The trouble with this metric is that the value of *LLE* depends not only on the model goodness-of-fit, but also on the number of trials, so it’s not clear how to interpret an arbitrary value of *LLE*, nor what magnitude of difference in *LLE* values constitutes a “meaningful” difference.

### Varying the number of free parameters

Although we cannot necessarily interpret an arbitrary value of *LLE*, we do know that larger values are better, signifying closer fit of model to data. One thing we *can* do is ask whether the model, as currently described, is the simplest possible description of the data: Can we do even better with more free parameters, or could we do nearly as well with fewer? What is the right level of complexity in our model?

For purposes of discussion, let us use the nickname DDM−5 to refer to our DDM with five free parameters: *a, z, d.s1, d.s2*, and *Ter*. The “best-fitting” parameters for DDM-5 (using MLE) were presented in Table 4, which also showed the maximal *LLE* obtained for each data file, using those “best-fitting” parameters.

We might then consider a DDM with only four free parameters: *a, z*, and *Ter* but only a single drift rate *d* to be used on all trials regardless of the stimulus. (In this case, we would likely assume a sign change: drift rate −*d* on s1 trials so that the evidence accumulation process tends downward to r1, and drift rate +*d* on s2 trials so that the evidence accumulation process tends upward to r2, but the magnitude of *d* does not differ for s1 and s2 trials, so it can be described by a single free parameter.) For purposes of discussion, let us call this version DDM-4 (DDM with four free parameters).

We could then conduct model-fitting on our dataset with DDM-4, just as we did with DDM-5. Assuming both the model-fitting process converges, and that the parameter estimates survive an initial sanity check, we could then compare the maximal *LLE* obtained under DDM-4 with that obtained DDM-5 (Table 6).

**Table 6:** Model comparison: Results of model-fitting with DDM-4; for ease of comparison, maximal LLE for each file under DDM-5 is also shown (reprinted from Table 4).

	Estimated parameters, using DDM-4					*LLE* (from DDM-4)	*LLE* (from DDM-5)
	*a*	*z*	*d.s1*	*d.s2*	*Ter*		
1	1.08	0.36	−1.87	1.87	0.22	150.6	185.2
2	1.88	0.22	−2.35	2.35	0.24	49.9	70
3	1.74	0.22	−2.36	2.36	0.15	69.2	128.5
4	1.17	0.43	−2.51	2.51	0.17	273.6	287.1
5	1.08	0.36	−1.88	1.88	0.22	153	189.7
6	1.65	0.3	−2.42	2.42	0.25	140.5	140.5
7	1.2	0.36	−2.02	2.02	0.21	129.5	129.5
8	0.78	0.51	−1	1	0.23	220.4	220.4
9	0.83	0.59	−2.01	2.01	0.18	319.4	331.9
10	0.85	0.47	−0.45	0.45	0.18	128.9	135.6
Mean	1.23	0.38	−1.89	1.89	0.2	163.5	181.8
SD	0.4	0.12	0.67	0.67	0.03	84.7	79.6

A first, important point is that the maximal *LLE* obtained under DDM-4 will (by definition) be less than or equal to that obtained by DDM-5. This is because any solution explored by DDM-4 (which constrains *d.s1* = −*d.s2*) should also be explored by DDM-5 (which allows the two drift rates to vary independently – not excepting those cases where they happen to have the same magnitude but different sign).

So, the question here is not whether DDM-4 can provide a better fit: we know that it cannot. The question is: can DDM-5 provide a *sufficiently* better fit than DDM-4, enough to justify its added complexity?

For example, Table 4 showed that the estimated parameters for participants #6, #7, and #8 under DDM-5 had drift rates *d.s1* and *d.s2* that were nearly equal in magnitude, though oppositely signed, and so DDM-4 (where the two drift rates are forced to have the same magnitude) provides just as large *LLE* as does DDM-5. And so, at least for these three participants, there does not appear to be much “advantage” to using the more complex model.

On the other hand, the larger DDM-5 provides a much better fit (larger *LLE*) for participants #1, #2 and #3. Averaged across all 10 participants, DDM-5 does provide numerically better mean *LLE* than DDM-4: 181.8 vs. 163.5. What we need is a way to quantify whether this 20-unit improvement in *LLE* is “significant” or “meaningful” – enough to justify our use of the more complex model.

### Is the more complex model “worth it”?

There are a number of metrics that can be used to address this question. One of the most commonly used is Akaike’s Information Criterion (AIC), which is an attempt to compare *LLE* between models while penalizing more complex models (Akaike, 1974): specifically, *AIC = 2 k* − 2*LLE, where *k* is the number of free parameters (5 for DDM-5 and 4 for DDM-4). The smaller *AIC*, the better; therefore, the addition of *2k* to the *LLE* results in a larger “penalty” (increasing AIC) for models with more free parameters. Using this formula, the mean AIC for DDM-5 is −354, and that for DDM-4 is −319, so we would conclude that the larger model, despite its added complexity, is a better description of the dataset.

A related metric, the Bayesian Information Criterion (BIC), considers number of parameters *k* as well as the number of trials *n* in the dataset (Schwartz, 1978): *BIC* = k*ln(n) − 2*LLE; again, lower (more negative) is better. BIC is only valid if *n* >> *k* (i.e., number of trials much larger than number of free parameters). A nice feature of BIC is that there are conventions for interpreting BIC values (Kass and Raftery, 1995): as a rule of thumb, if the difference in BIC between two models is <2, then the more complex model is “not worth it” (more formally, there is no positive evidence in favor of the more complex model, and so the simpler model should be preferred); a difference in BIC of >2 indicates positive evidence in favor of the more complex model, while BIC difference of >6 is considered strong evidence and >10 indicates very strong evidence in favor of the complex model.

In our example, DDM-5 has mean BIC of −333 and DDM-4 has mean BIC of −302. The difference is >30, so we conclude that there is very strong evidence favoring the more complex model with separate drift rates.

The above results assume that we used MLE as our model-fitting procedure. When Bayesian methods are used, AIC and BIC can be reported, but some articles instead report Deviance Information Criterion (DIC), which is a generalization of AIC for use when posterior distributions have been obtained *via* MCMC methods (Spiegelhalter et al., 2002), or the Watanabe-Akaike Information Criterion (WAIC; Watanabe, 2010), which is a generalized version of the AIC that can be used when the posterior distributions are not normal. In all cases, lower values indicate better fit after penalizing for model complexity.

If Bayesian methods have been used, it is also possible to report a Bayes Factor (BF), which is a ratio of the marginal likelihood of the two models, interpretable as the relative strength of the evidence that each model is correct. Values of BF = 1 mean the two models are equally likely, while larger values of BF make us increasingly confident in supporting the first hypothesis (or model). As a rule of thumb, BF > 3 is considered weak evidence and BF > 10 is considered strong evidence (e.g., Wagenmakers et al., 2018a).

All these metrics – AIC, BIC, DIC, WAIC, BF – are used to compare how well two models describe the same data file(s). As discussed earlier, there may be some participants for whom one model has a much lower metric than the other model, but some participants where the reverse is true. Often, a decision favoring one model or the other is based on a simple majority vote: which model results in best metrics for a majority of participants. Always, the burden is on the more complex model to justify its use, so if the complex model does not clearly provide a better fit, then the simpler model would likely be preferred.

And remember: model selection methods only tell us which of the models under consideration fits the data best. It does not guarantee that any of them are correct (nor that they are better than any of the infinitely many other models that might have been evaluated).

## Now (and only now), present the model results

So far, we have estimated best-fitting parameters for our data; we have conducted predictive checks and parameter recovery studies to assure ourselves that DDM-5 can accurately recover parameters for this type of data; and we have compared DDM-5 vs. DDM-4 (and possibly some other variations also), and concluded that DDM-5 is the best description of our data, providing excellent fits to the data with no unnecessary complexity (no more free parameters than are actually needed).

**Figure 10:**
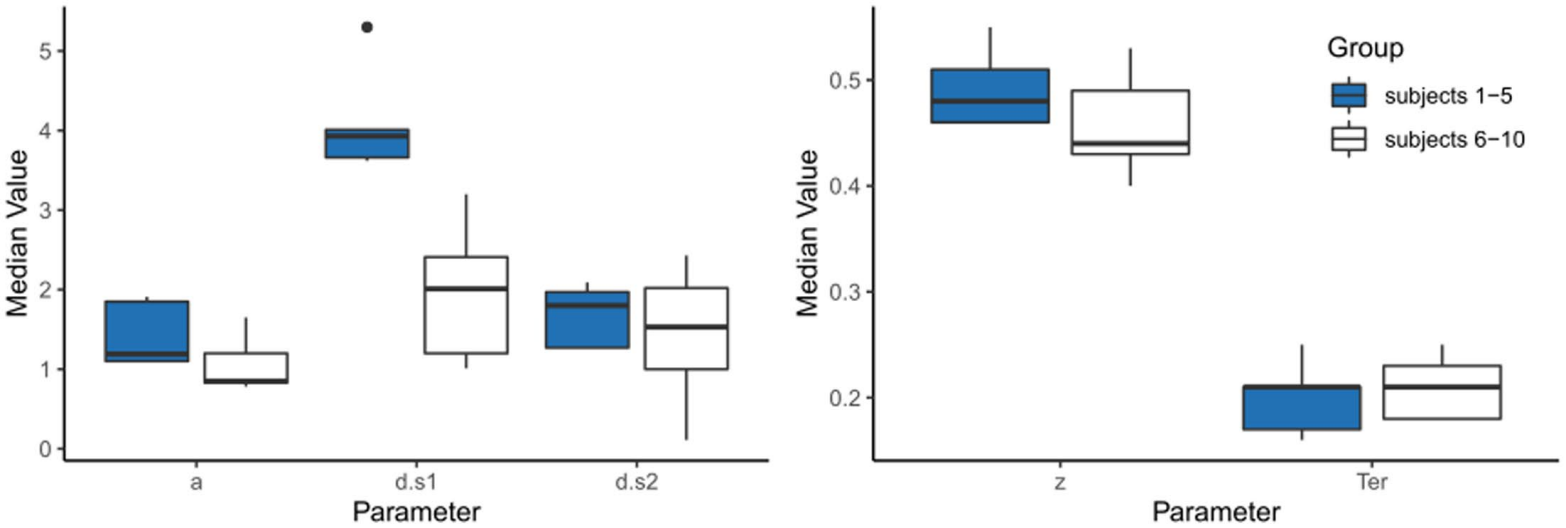
Box-and-whiskers plots showing DDM estimated parameter results from 10 data files, using MLE. **(Left)** Boundary separation a and drift rates d for s1 and s2, all in arbitrary units. For easy comparison, drift rates are plotted as absolute values; the actual value of *d.s1* < 0 reflects slope downward to r1 boundary while *d.s2* > 0 reflects slope upward to r2 boundary. **(Right)** Relative starting point *z* (as a proportion of a) and non-decision time Ter (in seconds). Heavy lines indicate median; boxes indicate interquartile range (IQR); whiskers indicate range; dots indicate scores lying >1.5 IQR beyond the box.

At this point, the parameter estimates from our DDM-5 can (finally!) be reported for individual data files and/or summarized for each group (e.g., patients vs. controls). These could be the value estimates for each parameter returned by MLE; or, if Bayesian methods were used, the results could be presented as medians or means of the posterior distributions for each parameter, or even plots of the posterior distributions for each parameter. For example, perhaps subjects #1–5 constitute the control group and subjects #6–10 constitute the experimental group in our study; Figure 10 plots the median (and IQR) parameter estimates for each group.

The point estimates for each parameter can be also subjected to statistical analysis, using analogous methods to those that were used to analyze the behavioral data (accuracy and RT). To illustrate, in our example study, it appears that *a, z,* and *Ter* are generally consistent across groups, but that one group has much higher drift rate for s1 than s2, whereas the other group has equivalent drift rates for the two stimulus types. This apparent interaction could be confirmed by ANOVA or other inferential statistics.

In the real world, we might be interested in comparing parameter estimates between patients vs. controls (e.g., higher response caution favoring accuracy over speed in schizophrenia; Moustafa et al., 2015) or across task conditions (e.g., mood induction shifts response bias in favor of mood-congruent responses; White et al., 2018a).

Additionally, just as with the original behavioral data, parameter estimates can be examined for relationships with other variables of interest (such as demographic, clinical, or neurocognitive variables). Some researchers have suggested that model parameters can be used as classifiers to distinguish patients from controls, and that addition of parameter estimates can improve classification beyond standard demographic and clinical variables and/or behavioral variables alone (e.g., Zhang et al., 2016; Myers et al., 2022 July 28).

## Reporting – And critically evaluating – The model

The final step in our study would be publishing the results and, as with any scientific method, it is important that the report be complete enough to allow the reader to critically evaluate the methods, the results, and the authors’ interpretation, including several key questions.

### What modeling approach was used, and was it appropriate for the job at hand?

Reporting requirements for the DDM should, at a minimum, state what free parameters were considered, and what method of model-fitting was used (e.g., *χ*^2^, MLE, Bayesian MCMC). Ideally, the authors should provide code for validation/replication. The reader should be able to evaluate whether the model design was appropriate for the cognitive task (and research hypothesis) under study; for example, is there an existing literature using these methods with the study population? If the authors present a new or modified approach, did they explain and justify this process?

For example, the “standard” DDM described here assumes rapid responding by well-trained participants, with little expectation that learning, practice, or fatigue will modify behavior across the study. Is this consistent with the behavioral task demands? Also, different models (and model-fitting methods) have different requirements, such as number of responses, number of total trials, and minimum number of correct/incorrect responses per trial type (see Table 2); were these requirements met? More generally, is the model likely to shed any light on the cognitive processes being investigated by the study hypothesis?

### Was the model validated?

Before presenting parameter estimates from the model, the authors should validate their model. First, did the authors present any theoretical justification for the free parameters being considered? Did they conduct predictive tests to show that the model can, in fact, generate simulated data that at least superficially captures important aspects of the behavioral data? Did they conduct a parameter recovery study to demonstrate that their model, as described, can accurately recover generating parameters? Did they conduct any model selection studies, to examine the effect of adding/deleting free parameters and did these results convincingly support the version of the model that the authors eventually reported?

Sometimes, this information is relegated to an appendix or an online supplement, but it is an important part of the modeling process, and the reader should be assured that this was done.

### Do the model results survive a sanity check?

Turning now to the model results, which are often parameter estimates (such as Figure 10 or Table 4), usually compared across one or more groups or task conditions: Do the reported results seem reasonable for this task, given what we know from prior studies in the literature? If violin plots or strip plots (or, for Bayesian methods, posterior distributions) are presented, are the results unimodal? Is there any evidence of floor/ceiling effects that might suggest a broader range of possible parameter values needs to be examined?

### Is there a thoughtful discussion of model limitations?

A good modeling paper (like all good science) will be honest about its limitations. At present, the DDM is almost always used *post-hoc*, rarely to test an *a priori* hypothesis. As such, overfitting is always a concern. Ideally, modeling results obtained in one sample should be validated in a new sample, but failing this (or until follow-up studies are conducted), authors can try techniques such as cross-validation (or “out-of-sample” testing), to fit the model to different subsets of the data and test how well it generalizes. At a bare minimum, the possibility of overfitting should be addressed when interpreting model results.

A second general limitation of DDM is that it can show that estimated parameters are *sufficient* to explain the empirical data; but can never *prove* that this is the case. Latent cognitive processes remain latent. Ideally, results from the model identify candidate cognitive processes that could then form the basis of future hypothesis-driven studies.

The DDM also makes many simplifying assumptions about the process of evidence accumulation and decision-making. Simplicity is a virtue, but of necessity leaves out complicating factors that can include variations in attention, emotion, and other processes that may influence decision-making. Standard versions of the models assume that the empirical RT distribution reflects a large number (dozens if not hundreds) of repeated measurements of a well-learned response under constant conditions. If the subject learns new response strategies, or loses attention, as the session proceeds, this assumption may not be valid. This issue is often partially remediated by having a long practice phase before “real” data collection starts, so that the RT and accuracy measurements reflect performance of a well-learned response.

### Perhaps most important: Does the model tell us anything non-trivial?

The main point of using computational models is (we hope) to uncover information about latent cognitive processes, and possibly to link these latent cognitive processes to brain substrates. So, did the current results actually provide any insights that would not be obvious from the behavior alone? For example, given that one group performed more slowly than another, can we understand this in terms of specific mechanisms such as increased boundary separation, reduced drift rate, and/or increased non-decision time – and if so, does this tell us anything interesting about the group in question? Even more interesting, can these parameters be mapped onto brain substrates or physiological processes?

Importantly, model results can sometimes be informative even in the presence of non-significant behavioral results. For example, even if two groups performed similarly in terms of accuracy and RT, perhaps the model can suggest qualitatively different ways in which the groups solved the speed-accuracy tradeoff (perhaps one group, with slower *Ter* due to motor dysfunction, “compensated” by reducing boundary separation).

Additionally, while group differences in model parameters can sometimes suggest important differences in underlying cognitive processes, absence of parameter differences can potentially show where a theory falls short, in failing to describe the phenomena of interest (Millner et al., 2020). In this way, the “failures” of a computational model can sometimes be as insightful as its successes.

## Conclusion

The above limitations notwithstanding, the DDM has become a dominant model of speeded decision-making, and some have argued that the DDM should replace mean RT and accuracy as default measurement tools for cognitive psychology (Evans and Wagenmakers, 2020). The DDM and other computational models are a useful complement to verbal theories in that they require explicit specification of cognitive components, and how these components interact (Millner et al., 2020). The idea of DDM parameters that correspond to fairly general (if latent) cognitive processes also aligns with RDoC (Research Domain Criteria), a research framework proposed by the U.S. National Institute of Mental Health (NIMH) for investigating mental disorders in the context of basic biological and cognitive processes that contribute to a range of neurobehavioral functions (Insel et al., 2010; Cuthbert and Insel, 2013).

The reader who has made it thus far will appreciate that using and understanding the DDM can take considerable investment of time, to understand the basic concepts, to acquire and master software, to design experiments that are consistent with planned modeling, and to interpret and report results. Yet, like other analysis methods in cognitive psychology and neuroscience, computational models can repay this investment by providing insightful and replicable results that complement standard behavioral measures. It is our hope that this article will help provide our colleagues in cognitive psychology and neuroscience with the background to appreciate and critically evaluate research articles that report modeling results, and even to consider using these computational models in their own research.

## Author contributions

CEM, AI, and AM contributed to the conceptualization, design, and writing of the manuscript. All authors contributed to the article and approved the submitted version.

## Funding

This work was funded by Merit Review Award #I01 CX001826 (CEM) from the U.S. Department of Veterans Affairs Clinical Sciences Research and Development Service. The funder had no role in study design, data collection and analysis, decision to publish, or preparation of the manuscript.

## Conflict of interest

The authors declare that the research was conducted in the absence of any commercial or financial relationships that could be construed as a potential conflict of interest.

## Publisher’s note

All claims expressed in this article are solely those of the authors and do not necessarily represent those of their affiliated organizations, or those of the publisher, the editors and the reviewers. Any product that may be evaluated in this article, or claim that may be made by its manufacturer, is not guaranteed or endorsed by the publisher.

## Appendix

### Supporting material

An R script file, used to generate the data-related figures and tables in this article, is provided online at https://osf.io/cpfzj/, along with the test data file (testdata.csv) and the 10 empirical data files (empdata1.csv…empdata10.csv) used to generate the results. All data files were generated for the purpose of this tutorial and do not constitute human subjects research data.

The R script was verified in October 2022 to run on a Macintosh iMac (Mac OS Catalina 10.15.7) running R version 4.1.2 (R Core Team, 2021), and a Dell PC (Windows Enterprise 10) running R version 4.1.1. The script should also run under other releases of R and on Linux machines; however, we do not maintain (and cannot guarantee) the R packages used in the script.

This code may be freely used and/or modified for users to run with their own datasets; however, we strongly suggest that users making their first foray into computational modeling carefully consult the extensive tutorials and documentation provided with the software packages (especially DMC, rtdists, and RWiener, used in the examples here), as well as familiarizing themselves with best practices for computational modeling (e.g., Daw, 2011; Heathcote et al., 2015; Wilson and Collins, 2019) and the many excellent DDM review articles available (some of which are cited in this article).
